# Predaceous water beetles (Coleoptera, Hydradephaga) of the Lake St Lucia system, South Africa: biodiversity, community ecology and conservation implications

**DOI:** 10.3897/zookeys.595.8614

**Published:** 2016-06-02

**Authors:** Renzo Perissinotto, Matthew S. Bird, David T. Bilton

**Affiliations:** 1 DST/NRF Research Chair in Shallow Water Ecosystems, Nelson Mandela Metropolitan University, C/o Department of Zoology, P.O. Box 77000, Port Elizabeth, 6031, South Africa; 2 Marine Biology and Ecology Research Centre, School of Marine Science & Engineering, Plymouth University, Drake Circus, Plymouth PL4 8AA, United Kingdom

**Keywords:** Aquatic beetles, biodiversity census, Afrotropical region, wetland conservation, iSimangaliso Wetland Park

## Abstract

Water beetles are one of the dominant macroinvertebrate groups in inland waters and are excellent ecological indicators, reflecting both the diversity and composition of the wider aquatic community. The predaceous water beetles (Hydradephaga) make up around one-third of known aquatic Coleoptera and, as predators, are a key group in the functioning of many aquatic habitats. Despite being relatively well-known taxonomically, ecological studies of these insects in tropical and subtropical systems remain rare. A dedicated survey of the hydradephagan beetles of the Lake St Lucia wetlands (South Africa) was undertaken between 2013 and 2015, providing the first biodiversity census for this important aquatic group in the iSimangaliso Wetland Park, a UNESCO World Heritage Site within the Maputaland biodiversity hotspot. A total of 32 sites covering the entire spectrum of waterbody types were sampled over the course of three collecting trips. The Lake St Lucia wetlands support at least 68 species of Hydradephaga, a very high level of diversity comparing favourably with other hotspots on the African continent and elsewhere in the world and a number of taxa are reported for South Africa for the first time. This beetle assemblage is dominated by relatively widespread Afrotropical taxa, with few locally endemic species, supporting earlier observations that hotspots of species richness and centres of endemism are not always coincident. Although there was no significant difference in the number of species supported by the various waterbody types sampled, sites with the highest species richness were mostly temporary depression wetlands. This contrasts markedly with the distribution of other taxa in the same system, such as molluscs and dragonflies, which are most diverse in permanent waters. Our study is the first to highlight the importance of temporary depression wetlands and emphasises the need to maintain a variety of wetland habitats for aquatic conservation in this biodiverse region.

## Introduction

The aquatic Adephaga, or Hydradephaga, with over 5300 species currently described worldwide, account for around one third of the total aquatic and semi-aquatic beetles described to date (Lancaster and Downes 2013). The group is dominated by the diving beetles (Dytiscidae), with over 4300 species globally (Nilsson 2015), but also includes the familiar whirligig beetles (Gyrinidae), the crawling water beetles (Haliplidae) and the burrowing water beetles (Noteridae), as well as a number of smaller families with largely relictual distributions (Shull et al. 2001, Beutel et al. 2005, Stals and de Moor 2007, Jäch and Balke 2008, Toussaint et al. 2016).

Hydradephaga are important predators in aquatic systems, consuming a wide variety of benthic and pelagic invertebrates, mainly other insects and crustaceans (Beutel and Leschen 2005, Lancaster and Downes 2013, Culler et al. 2014). Due to their aerial respiration and winged adults, many species are able to fly long distances and move between waterbodies in response to, for example, seasonal drought – a strategy most common in the inhabitants of small, temporary lentic waters (Bilton 2014a). Like most aquatic beetles, Hydradephaga are primarily freshwater insects, but a number of species are adapted to high salinities and may dominate the macroinvertebrate fauna of hypersaline inland waters (Stals and de Moor 2007, Millan et al. 2011, Lancaster and Downes 2013); indeed as a group, the aquatic Adephaga are found across the entire spectrum of inland waters, making them an excellent focal taxon in freshwater ecology and conservation (Foster and Bilton 2014). Hydradephaga, and indeed water beetles in general, are excellent surrogates of wider freshwater macroinvertebrate biodiversity, their assemblages reflecting not only overall species richness, but also patterns of community composition very well (Bilton et al. 2006, Sánchez-Fernández et al. 2006, Guareschi et al. 2015). Water beetles are diverse both ecologically and in terms of life-history (Jäch and Balke 2008), and are functionally important in most inland waters, being involved in a range of ecosystem processes such as nutrient cycling and processing. They have been used to select priority areas for aquatic conservation in a number of countries and regions (e.g. Foster et al. 1989, Sánchez-Fernández et al. 2004, Foster and Bilton 2014), but despite excellent global or regional catalogues (e.g. Nilsson 2001, Nilsson and van Vondel 2005) this is hampered in many areas by a lack of baseline ecological data, particularly in the Afrotropics.

Lake St Lucia on the north-east coast of South Africa is a prominent coastal system, historically communicating freely with the open ocean most of the time. Recently, freshwater deprivation related to anthropogenic manipulation and a regional drought has led to large-scale desiccation and closure of its mouth since July 2002 (Whitfield and Taylor 2009, Perissinotto et al. 2013). However, climatic conditions in the area have changed, with the establishment of a new wet phase during the period 2010 - 2014. This has resulted in repeated flood events, with large amounts of fresh water flowing into the system and changing the prevailing salinity state from hypersaline to oligo- or polyhaline.

Lake St Lucia itself sits within the iSimangaliso Wetland Park, South Africa’s first UNESCO World Heritage Site, and a Ramsar wetland of global significance due to its exceptional biodiversity. iSimangaliso and the coastal plains of KwaZulu-Natal form part of Maputaland, a centre of endemism and transition zone between tropical lowlands to the north and temperate regions to the south and west. Warm currents flowing south from Mozambique mean that the region’s biota is now dominated by tropical species at what is a relatively high southern latitude (van Wyk and Smith 2001, Connell et al. 2013).

A detailed biodiversity census has already been completed for some prominent invertebrate groups at St Lucia/iSimangaliso, including the bivalves (Nel et al. 2013), gastropods (Perissinotto et al. 2014) and true crabs (Peer et al. 2014). Historically, there has been little dedicated work on the aquatic insects of the park, with the exception of opportunistic collections (some now deposited in museums) and a few publications in the grey literature (e.g. Vrdoljak 2004). An extensive study of the Odonata has been completed recently (Hart et al. 2014), however, revealing a rich fauna at iSimangaliso. As preliminary investigations suggested high aquatic beetle diversity in the park, the current study was undertaken to provide a better understanding of aquatic beetle biodiversity in this globally important region. Here we provide records for all hydradephagan species recorded during dedicated surveys at iSimangaliso, together with multivariate analyses of predaceous water beetle assemblage composition and a brief review of historical records. Given the general lack of such data from southern Africa, and the Afrotropical region in general, our study provides a valuable baseline for the study of this key group of freshwater macroinvertebrates in southern Africa.

## Methods

### Study area

**Figure 1.**
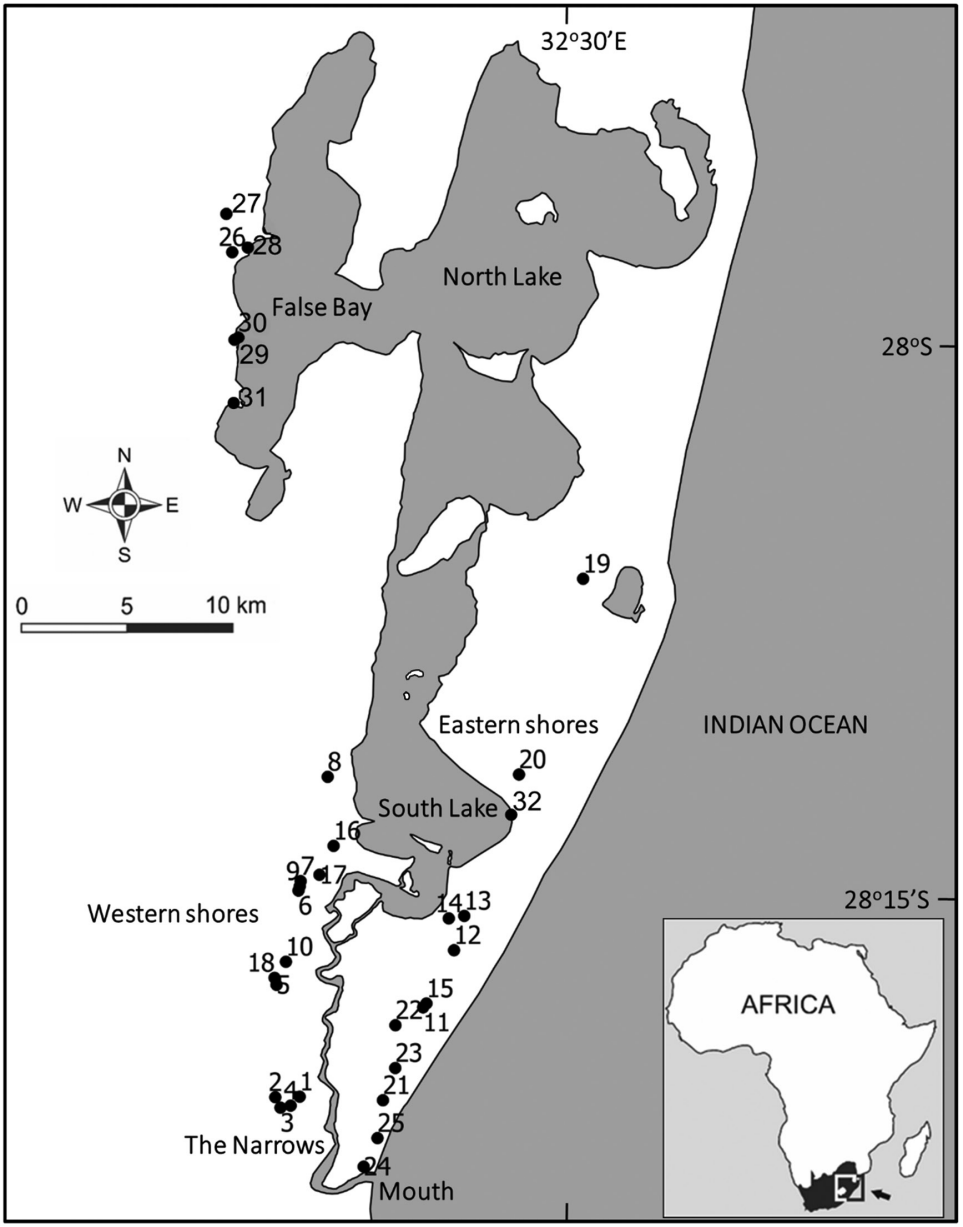
The Lake St Lucia system in northern KwaZulu-Natal. The locations of sites sampled between November 2013 and February 2015 are depicted. Site numbers 1–32 correspond to those in Table 1.

Samples were collected in and around Lake St Lucia (27°52'0"S to 28°24'0"S and 32°21'0"E to 32°34'0"E), a large (~ 300 to 350 km^2^) estuarine lake in northern KwaZulu-Natal, South Africa (Fig. 1). The St Lucia system comprises three interconnected shallow lakes (South Lake, North Lake and False Bay) that are linked to the Indian Ocean via a narrow channel (known as the Narrows) of 21 km in length (Fig. 1). Dedicated surveys of the aquatic beetles of Lake St Lucia and its associated wetlands were undertaken during November 2013 (19^th^–30^th^), July 2014 (23^rd^–24^th^) and January-February 2015 (31^st^ January–6^th^ February).

Aquatic beetles were collected from a variety of freshwater habitats surrounding Lake St Lucia and from submerged vegetation habitats at the margins of the estuary itself. A total of 32 sites comprising a wide variety of waterbodies was sampled over the course of the three collection trips (Fig. 1). Sites were chosen in an attempt to cover the range of wetland habitats present in the region, and ranged from open water in the lake itself to the small forest pools and seepages. They also covered most of the geographical area contained within the St Lucia section of the iSimangaliso Wetland Park. Parts of the western shore (lack of freshwater habitats) and the entire northern and north-eastern shore of the lake (inaccessibility) were not covered by this study.

**Figure 2.**
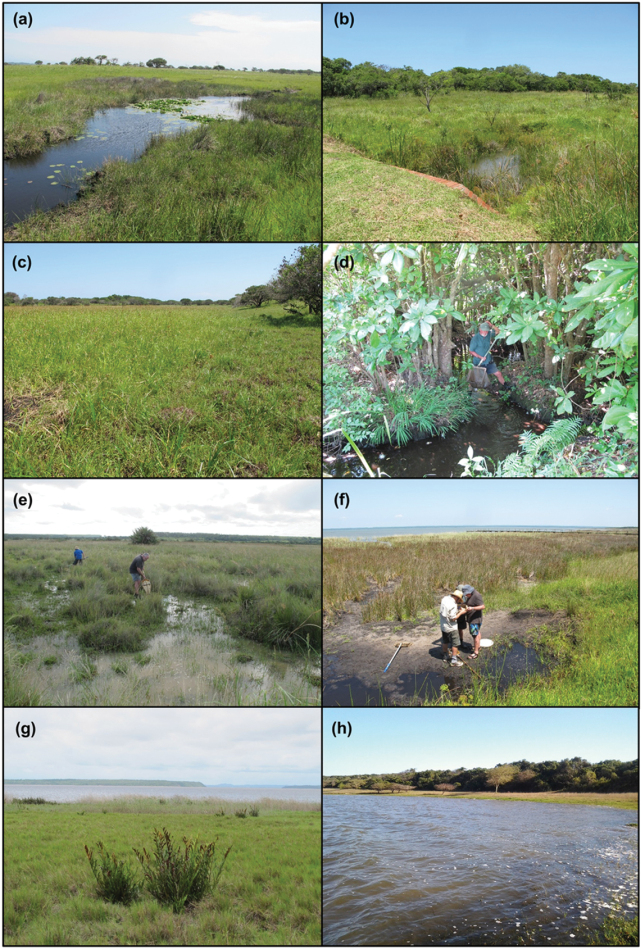
Examples of the wetland habitat types encountered in the St Lucia section of the iSimangaliso Wetland Park during the course of this study. **a** Depression (site 13) **b** valley bottom (channelled, site 17) **c** valley bottom (unchannelled, site 19) **d** river (site 20) **e** wetland flat (site 18) **f** seepage (site 32) **g** estuarine lake shore (site 31) **h** estuarine lake body (site 30).

In addition to sampling of the estuarine lake itself (Fig. 2g–h), the following wetland habitats were sampled (following the classification scheme of Ollis et al. 2015): depression wetlands (both isolated and non-isolated, Fig. 2a); valley bottom wetlands (both channelled and un-channelled, Fig. 2b–c); rivers (both in-channel and riparian habitats, Fig. 2d); a wetland flat (Fig. 2e); and a seepage wetland (Fig. 2f). The most frequently encountered habitat was depression wetlands (n = 16), followed by valley bottom wetlands (n = 8), rivers (n = 4), with a single wetland seep and a single wetland flat. Virtually all habitats encountered in this study were extensively vegetated by a mix of emergent and submerged macrophytes. Although the exact inundation regime is not known for each of the waterbodies, some of the smaller depression and valley bottom wetlands are expected to be temporary systems. This was confirmed during the January-February 2015 sampling expedition, when several of the sites sampled previously were dry. Table 1 provides a summary of the locations sampled, their habitat classification and dates of sampling.

**Table 1.** Geographic position and classification of the waterbodies sampled during this study. Sampling took place during the three collecting trips to Lake St Lucia during November 2013, July 2014 and January/February 2015. Classification (wetland type) follows the hydrogeomorphic (HGM) approach of Ollis et al. (2015). WS – Western Shores; ES – Eastern Shores; FB – False Bay.

Site	GPS (D°M’S”)		Wetland type	Region	November 2013	July 2014	January/February 2015
1	28°20'53.33"S	32°23'38.42"E	River (pool)	WS		x	x
2	28°20'54.23"S	32°22'59.68"E	Depression	WS		x	
3	28°21'10.77"S	32°23'7.88"E	Channelled valley bottom	WS		x	
4	28°21'7.52"S	32°23'24.04"E	Channelled valley bottom	WS		x	
5	28°17'55.76"S	32°23'10.62"E	River (riparian zone)	WS		x	
6	28°15'26.06"S	32°23'36.51"E	Depression	WS	x	x	x
7	28°15'11.10"S	32°23'39.95"E	Depression	WS	x	x	x
8	28°12'25.44"S	32°24'22.97"E	Depression (artificial)	WS		x	
9	28°15'19.19"S	32°23'38.53"E	Depression	WS		x	
10	28°17'19.08"S	32°23'16.53"E	Depression	WS		x	
11	28°18'31.52"S	32°26'54.54"E	Un-channelled valley bottom	ES		x	
12	28°17'00.81"S	32°27'43.78"E	Depression	ES		x	
13	28°16'6.26"S	32°28'00.02"E	Depression	ES		x	x
14	28°16'10.26"S	32°27'35.43"E	Depression	ES		x	x
15	28°18'25.29"S	32°26'59.88"E	Un-channelled valley bottom	ES		x	
16	28°14'15.05"S	32°24'32.30"E	Depression	WS			x
17	28°15'1.00"S	32°24'9.85"E	Channelled valley bottom	WS			x
18	28°17'44.59"S	32°22'58.49"E	Flat	WS			x
19	28°07'10.99"S	32°31'8.98"E	Un-channelled valley bottom	ES			x
20	28°12'21.75"S	32°29'27.07"E	River (main channel)	ES			x
21	28°20'59.06"S	32°25'50.76"E	Depression	ES			x
22	28°18'59.92"S	32°26'10.64"E	Depression	ES			x
23	28°20'7.84"S	32°26'10.36"E	Depression	ES			x
24	28°22'44.46"S	32°25'20.13"E	River (connected to estuary)	ES			x
25	28°21'59.12"S	32°25'42.10"E	Depression	ES			x
26	27°58'32.33"S	32°21'51.14"E	Depression	FB			x
27	27°57'31.50"S	32°21'41.82"E	Depression	FB	x		x
28	27°58'25.01"S	32°22'16.02"E	Channelled valley bottom	FB			x
29	28°00'51.44"S	32°21'54.93"E	Channelled valley bottom	FB	x		x
30	28°00'47.95"S	32°22'00.92"E	Estuarine lake	FB	x		x
31	28°02'9.17"S	32°21'42.78"E	Estuarine lake shore (light trap)	FB	x	x	x
32	28°13'14.56"S	32°29'12.45"E	Seep	ES			x

### Field sampling protocol

Sweep netting was employed on all three sampling trips as the primary method for collecting aquatic beetles. A long-handled square-framed sweep net with a 30 cm mouth and 1 mm mesh was swept repeatedly from the water surface to the bottom substrate and back to the surface following a protocol similar to that of Bilton et al. (2006) and Bird et al. (2013). Sweep netting effort was concentrated in submerged vegetation and around shore margins. Sampling was not quantitative, but for most waterbodies approximately 20 sweeps were performed (semi-quantitative sampling). In addition to sweep netting, the margins of each waterbody were searched visually for shore beetles and semi-aquatic taxa. A light trap, consisting of a 4x3 m white sheet hung vertically below a fluorescent mercury vapour lamp (Radiant 250 W), was deployed on all three survey trips at False Bay, adjacent to the lake shore, during the evening. Aquatic coleopteran specimens were retrieved from the light sheet by hand. All beetle specimens collected during the November 2013 and July 2014 surveys were killed using ethyl acetate vapour and preserved in 5% formalin solution. Specimens collected during January-February 2015 were preserved in 70% ethanol.

To provide an environmental context for the beetle samples and baseline information for the aquatic habitats of St Lucia, basic *in situ* physico-chemical parameters were measured at each site. Temperature, conductivity, salinity, pH, turbidity and dissolved oxygen were recorded using a YSI 6600-V2 multi-system probe. Due to technical problems, physico-chemical measurements were not taken during November 2013.

### Historical records and ad hoc collections

Records of aquatic Coleoptera previously collected from Lake St Lucia and the fresh waterbodies in its immediate vicinity were obtained from the Iziko South African Museum
(ISAM, Cape Town), the Ditsong National Museum of Natural History
(DNMNH, Pretoria; formerly the Transvaal Museum) and the South African National Collection of Insects
(SANC, Pretoria). Other data on aquatic beetle species collected historically in the St Lucia area were extracted from the works of Biström and Nilsson (2002), Biström et al. (2015), Day et al. (1954), Millard and Broekhuysen (1970) and Vrdoljak (2004). Some specimens were also obtained during opportunistic collections undertaken by the authors during 2008 and 2012. With the exception of species in Biström and Nilsson (2002) and Biström et al. (2015), specimens have not been examined by hydradephagan specialists, meaning that identifications of historical material may be inaccurate in some cases. Apart from species which do not occur in southern Africa (see below), these records are presented here for the sake of completeness, but should be viewed with caution.

### Identification and illustrations

All identifications were conducted by DTB, using a wide range of literature and, in many cases, comparison with named museum material. All identifications were based, at least in part, on the study of male genitalia, unless otherwise stated. Digital photographs of both ventral and dorsal habitus of each species were taken using a Canon Powershot G11 or a Canon EOS 600D digital camera fitted with a Sigma 50mm f/2.8 EX DG macro lens for larger specimens (≥ 1.5 cm) and a Nikon SMZ25 microscope for smaller specimens (< 1.5 cm). Image stacks were produced by hand, and combined using Zerene Stacker software (www.zerenesystems.com). Photographs were then compiled into an annotated and illustrated species list of all Hydradephaga identified within the November 2013, July 2014 and January/February 2015 surveys, as well as those collected from 2008 and 2012 during ad hoc collections (Appendix 1).

### Statistical analysis

Median and range values for each of the physico-chemical variables were calculated for each survey. The measured physico-chemical variables were then explored using multivariate analyses to assess how the physico-chemistry of freshwater wetlands at St Lucia varies amongst waterbody types and also spatially across the coastal plain. Principal Component Analysis (PCA) was used to depict patterns in physico-chemistry on a two-dimensional plot. Variables with non-normal distributions were log-transformed where appropriate. Conductivity was not depicted in the PCA plot as it was highly collinear with salinity (r = 0.996) and thus we regarded conductivity as a redundant variable.

Spatial patterns in the composition of aquatic beetle communities amongst the wetlands at St Lucia were analysed using multivariate techniques. Beetle presence-absence data were converted to a Bray-Curtis dissimilarity matrix and depicted on a two-dimensional plot using non-metric multidimensional scaling (MDS). Permutational MANOVA (PERMANOVA, Anderson 2001) was used to test for differences in beetle assemblage composition amongst the different regions of St Lucia (eastern shores, western shores and False Bay) and amongst different waterbody types (excluding seeps and flats, for which only one site each was sampled), once again using a Bray-Curtis dissimilarity matrix. Species richness (number of species recorded per waterbody) was similarly compared amongst regions and waterbody types, but in this case, given the univariate data, the Kruskal-Wallis non-parametric ANOVA approach was used.

All tests were performed using an *a priori* significance level of α = 0.05. PCA and MDS were performed using PRIMER v6 software (Clarke and Warwick 2001, Clarke and Gorley 2006). Permutational MANOVA was performed using the PERMANOVA routine in the PERMANOVA+ add-on package (Anderson et al. 2008) to PRIMER v6. P-values for PERMANOVA models were tested using 9999 unrestricted permutations of the raw data. Univariate techniques for species richness analyses were implemented using Statistica 12 software for Windows (Statsoft Inc. 2015).

## Results

### Physico-chemical characteristics of St Lucia waterbodies

Waterbodies encountered on the St Lucia coastal plain were mostly groundwater-fed depressions and valley bottom wetlands. These freshwater wetlands appeared to be abundant in the study area. True rivers (flow contained within a single main channel) were less common, but several small rivers were encountered and sampled (e.g. Nkazana Stream, site 20). Most waterbodies were small (generally < 2 ha) and shallow (<1 m maximum depth), although some of the valley bottom wetland sites formed part of larger systems (e.g. site 19 forms part of the Mfabeni mire). Due to a general predominance of lentic or slow-flowing systems, rocky biotopes were virtually absent and sites were extensively vegetated by a mix of emergent and submerged macrophytes, which formed the primary structural habitat for all sites sampled.

Surface water temperatures were warm and in summer the median recorded water temperature was as high as ~29 °C (Table 2). Sites were generally fresh with median conductivity and salinity values all below 1 mS.cm^-1^ and 0.5 PSU respectively (Table 2). The exception to this was for sites with a direct connection to the estuarine lake (see Table 1), which, not surprisingly, displayed elevated salinity. Median pH values (Table 2), were close to neutral, but there was substantial variation around the medians and sites ranged from alkaline (e.g. sites 1, 25 and 30) to highly acidic (e.g. sites 4 and 7). Turbidity was extremely variable across sites, waterbodies ranging from very clear (< 10 NTU, e.g. sites 7 and 14) to highly turbid (> 1000 NTU, e.g. sites 10 and 26). Median turbidity values suggest, however, that most sites were moderately turbid (75.8 and 30.2 NTU for the winter and summer sampling expeditions respectively, Table 2). Dissolved oxygen levels were elevated in summer compared to winter (median values of 7.14 and 4.53 mg.L^-1^ respectively, Table 2).

**Table 2.** Physico-chemical variables recorded during the July 2014 and January/February 2015 surveys. Median, minimum and maximum values are reported for each survey. Physico-chemical data were not collected in November 2013. Depth was not recorded in July 2014. Site 31 is not reported as this was a terrestrial light trapping location.

Survey date	Site	Temperature (°C)	Conductivity (mS.cm^-1^)	Salinity (PSU)	pH	Turbidity (NTU)	Dissolved O_2_ (mg.L^-1^)	Depth (m)
July 2014	1	16.59	0.254	0.14	8.61	16.4	4.72	-
	2	16.08	0.471	0.29	6.81	883.6	5.15	-
	3	16.50	0.601	0.35	6.58	194.8	1.81	-
	4	17.08	0.153	0.09	4.60	570.5	1.40	-
	5	17.03	6.726	4.42	6.56	40.2	2.45	-
	6	19.07	0.437	0.24	7.07	831.3	1.61	-
	7	22.81	0.504	0.25	5.22	75.8	3.58	-
	8	21.65	0.475	0.24	7.00	529.1	8.50	-
	9	21.02	0.489	0.26	7.23	183.2	7.88	-
	10	19.17	0.943	0.53	7.33	1220.2	7.83	-
	11	16.98	0.104	0.05	5.97	15.0	3.20	-
	12	22.62	0.221	0.10	6.46	36.7	10.05	-
	13	23.02	0.344	0.16	6.52	11.2	8.08	-
	14	21.96	0.240	0.11	6.60	3.2	4.53	-
	15	19.38	0.214	0.10	5.44	47.0	2.99	-
	Median	19.17	0.437	0.24	6.58	75.8	4.53	-
	Minimum	16.08	0.104	0.05	4.60	3.2	1.40	-
	Maximum	23.02	6.726	4.42	8.61	1220.2	10.05	-
January / February 2015	1	24.02	0.378	0.18	6.62	9.6	1.00	0.34
	6	34.37	1.034	0.50	5.06	30.2	10.23	0.31
	7	32.93	0.923	0.45	3.88	6.1	7.52	0.43
	13	22.47	0.592	0.28	5.69	197.6	4.39	0.38
	14	35.61	0.342	0.16	6.89	29.4	9.92	0.27
	16	31.87	2.551	1.29	7.57	613	3.58	0.24
	17	24.57	1.866	0.95	4.22	21.8	4.23	0.31
January / February 2015	18	34.23	0.237	0.11	7.32	191.2	7.42	0.26
	19	37.78	0.392	0.18	5.49	18.9	7.25	0.25
	20	24.33	0.411	0.20	5.96	34.8	1.27	0.37
	21	33.28	0.180	0.08	6.77	352.3	7.26	0.20
	22	34.73	0.827	0.40	4.82	25.6	8.13	0.50
	23	34.66	0.414	0.19	5.96	36.6	8.76	0.30
	24	25.84	9.752	5.48	7.65	25.8	4.32	0.70
	25	29.03	1.703	0.85	8.28	17.4	8.95	2.00
	26	26.09	0.323	0.15	7.21	1310.5	1.50	0.08
	27	22.03	0.598	0.29	7.42	151	1.82	0.70
	28	28.69	20.150	11.97	7.98	30.6	9.82	0.15
	29	26.73	1.496	0.75	6.90	306.3	5.53	0.25
	30	22.03	46.640	30.31	8.51	14.2	7.14	0.12
	32	30.03	0.710	0.35	7.55	14.6	6.98	0.05
	Median	29.03	0.710	0.35	6.89	30.2	7.14	0.30
	Minimum	22.03	0.180	0.08	3.88	6.1	1.00	0.05
	Maximum	37.78	46.640	30.31	8.51	1310.5	10.23	2.00

**Figure 3.**
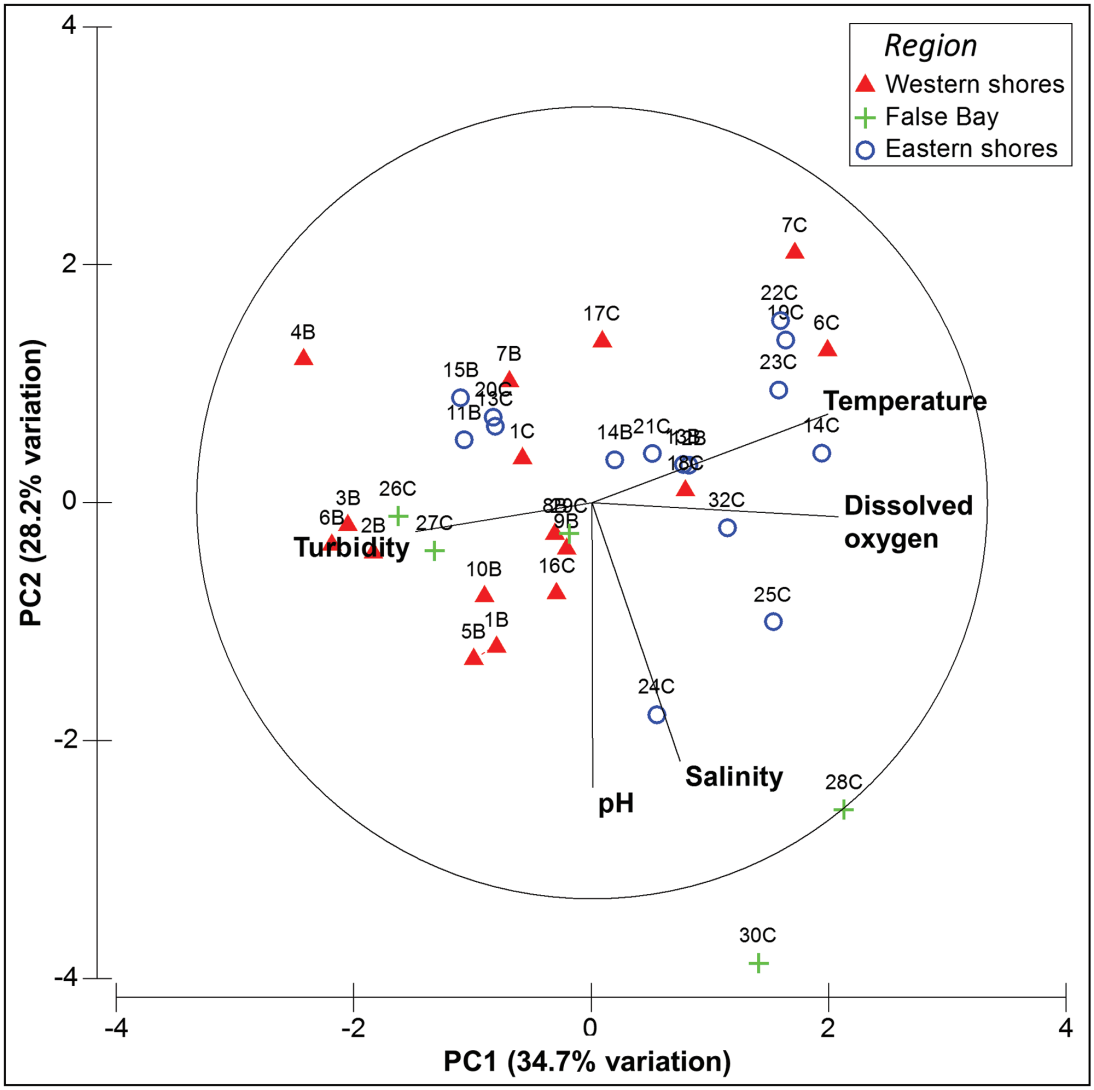
Principal components analysis (PCA) of physico-chemical variables. Sites are shown according to region and site numbers coded as B (July 2014) or C (January/February 2015). Physico-chemical data were not collected during the first survey (November 2013). The first two principal component axes are displayed, with PC1 and PC2 explaining 34.7 and 28.2% of the variation in the physico-chemical data, respectively. Table 2 provides the units in which each of the variables was measured.

The PCA plot (Fig. 3) offers a two-dimensional depiction of the relative positioning of sites according to their physico-chemical characteristics. The waterbodies sampled varied substantially in their overall physico-chemistry, spanning clear gradients of turbidity, temperature and dissolved oxygen, but less so for salinity, with most of the sites being fresh. There was some separation of sites according to season of sampling, with sites sampled in July 2014 (coded ‘B’ in Fig. 3) generally occurring to the left of the plot and sites sampled in January/February 2015 (coded ‘C’ in Fig. 3) generally occurring towards the right. Waterbodies across the three regions sampled (eastern shores, western shores and False Bay, see Table 1) showed some differentiation (Fig. 3), with most False Bay sites falling towards the left of the plot, although the overall pattern of differentiation was weaker and there is considerable overlap in physico-chemistry between some individual sites (e.g. sites 7 and 15).

### Aquatic beetles

Appendix 1 provides an illustrated checklist of the hydradephagan beetle taxa collected at St Lucia during the course of this study. Sixty-eight taxa were identified across the three sampling occasions (Table 3). Of these, 67 taxa were identified to species level, and one (*Peltodytes* sp., represented only by females) to genus. Of the beetles identified during our surveys, two belonged to the Gyrinidae, two to the Haliplidae, 12 to the Noteridae and 52 to the Dytiscidae. Thus dytiscids overwhelmingly dominated the hydradephagan species richness at St Lucia, as they do worldwide.

**Table 3.** Hydradephagan beetles collected from St Lucia during the course of this study. The sites are listed from which each taxon was collected on each of the three sampling trips. Site numbers 1–32 correspond to those listed in Table 1. The regions where each taxon occurred are also indicated: WS – western shores; ES – eastern shores; FB – False Bay. Species new to South Africa are shown in bold type.

	Sampling date			Region		
	November 2013	July 2014	January/February 2015	WS	ES	FB
Gyrinidae:						
*Gyrinus natalensis* Régimbart, 1892		4		X		
*Dineutus subspinosus* (Klug, 1834)			21, 23, 29		X	X
Haliplidae:						
*Haliplus natalensis* Wehncke, 1880			27			X
*Peltodytes* sp.			6, 27	X		X
Noteridae:						
*Canthydrus apicicornis* Régimbart, 1895		7, 8, 13	6, 7, 13, 14, 17, 18, 22, 25	X	X	
*Canthydrus marshalli* Balfour-Browne, 1939			6	X		
*Canthydrus notula* (Erichson, 1843)		6, 7, 8, 9, 10, 14, 15	6, 7, 14, 16, 17, 21, 22, 23, 27, 29	X	X	X
*Canthydrus quadrivittatus* (Boheman, 1848)		13	1, 6, 13, 14, 16, 17, 18, 22, 23, 27	X	X	X
*Canthydrus sedilloti* Régimbart, 1895			1, 6, 13, 14, 16, 17, 22, 23	X	X	
***Hydrocanthus grandis* (Laporte, 1835)**			14		X	
*Hydrocanthus micans* Wehncke, 1883			1, 6, 14, 17, 22, 23, 29	X	X	X
*Hydrocanthus ferruginicollis* Régimbart, 1895	27					X
*Synchortus imbricatus* (Klug, 1853)			1, 7, 16, 17, 25,	X	X	
***Synchortus desaegeri* Gschwendtner, 1935**			23		X	
*Neohydrocoptus aethiopicus* (Balfour-Browne, 1961)		2	1, 7, 20, 23, 27	X	X	X
*Neohydrocoptus angolensis* (Peschet, 1925)		1, 2, 3	1, 6, 14, 25, 27	X	X	X
Dytiscidae:						
+Copelatus cf. ejactus Omer-Cooper, 1965			17, 18, 22, 26, 29	X	X	X
*Copelatus erichsoni* Guérin-Méneville, 1849			17, 20, 26, 27, 29	X	X	X
*Copelatus pulchellus* (Klug, 1834)			17, 26, 29		X	X
*Cybister gschwendtneri* Guignot, 1935	27, 31		14, 27		X	X
*Cybister marginicollis* Boheman, 1848	27, 31	11	7, 14, 18, 22, 27, 31	X	X	X
*Cybister natalensis* (Wehncke, 1876)			29			X
*Cybister senegalensis* Aubé. 1838			6, 27	X		X
*Cybister tripunctatus africanus* Laporte, 1835			14, 21, 27, 29, 31		X	X
*Cybister bimaculatus* Aubé, 1838			21		X	
***Cybister ertli* Zimmermann, 1917**		13	14		X	
*Cybister vicinus* Zimmermann, 1917			21, 23, 27, 29, 31		X	X
*Cybister vulneratus* Klug, 1834	30	4	6, 14, 16, 17, 18, 19, 23, 27, 29, 31	X	X	X
*Rhantaticus congestus* (Klug, 1833)	27		21, 22, 23, 27, 29, 31		X	X
*Eretes sticticus* (Linnaeus, 1767)			21		X	
*Hydaticus bivittatus* Laporte, 1835			27			X
*Hydaticus exclamationis* Aubé, 1838			27, 29			X
*Hydaticus flavolineatus* Boheman, 1848			27			X
+Hydaticus cf. natalensis Guignot, 1951			27			X
*Hydaticus matruelis* Clark, 1864			27			X
*Hydaticus servillianus* Aubé, 1838	27, 30	6, 11	14, 23, 26, 27, 29	X	X	X
*Bidessus sharpi* Régimbart, 1895			6, 14, 16, 21, 27, 29	X	X	X
*Clypeodytes meridionalis* Régimbart, 1895		3	14, 20	X	X	
*Hydroglyphus farquharensis* (Scott, 1912)			6, 14, 16, 17, 18, 21, 22, 27, 28, 29, 31	X	X	X
*Hydroglyphus lineolatus* (Boheman, 1848)			21, 31		X	X
*Hydroglyphus zanz ibarensis* (Régimbart, 1906)		2, 3, 10	6, 13, 14, 16, 17, 21, 22, 29, 32	X	X	X
*Leiodytes hieroglyphicus* (Régimbart, 1894)			7, 17	X	X	
*Pseuduvarus viticollis* (Boheman, 1848)			32		X	
*Uvarus gschwendtneri* (Guignot, 1942)			6, 17, 18, 22, 23, 27, 29	X	X	X
*Hydrovatus acuminatus* Motschulsky, 1859			1, 6, 13, 14, 16, 22, 23, 25, 27, 29	X	X	X
*Hydrovatus cribratus* Sharp, 1882			6, 7, 14, 16	X	X	
*Hydrovatus dentatus* Bilardo & Rocchi, 1990			14, 17, 21	X	X	
***Hydrovatus eximius* Biström, 1997**			29			X
*Hydrovatus nefandus* Omer-Cooper, 1957			23, 27		X	X
*Hydrovatus nigricans* Sharp, 1882			22, 23		X	
*Hydrovatus oblongipennis* Régimbart, 1895		15	23		X	
*Hydrovatus obsoletus* Peschet, 1922			23		X	
*Hydrovatus villiersi* Guignot, 1955			14		X	
***Hydrovatus visendus* Biström, 1997**			6, 18	X		
*Herophydrus guineensis* (Aubé, 1838)			6, 7, 14, 16, 21, 27, 29	X	X	X
*Herophydrus inquinatus* (Boheman, 1848)	27		27, 29			X
+*Herophydrus nigrescens* Biström & Nilsson, 2002			6, 25, 27	X	X	X
*Herophydrus nodieri* (Régimbart, 1895)	27	6, 11, 12	6, 7, 13, 14, 22, 23, 25, 27, 29	X	X	X
*Heterhydrus senegalensis* (Laporte, 1835)			6, 14, 22, 23	X	X	
*Hyphydrus caffer* Boheman, 1848			14, 25		X	
*Hyphydrus cycloides* Régimbart, 1889			14, 18, 22, 23, 29	X	X	X
*Hyphydrus signatus* Sharp, 1882	27		6, 27, 29	X		X
*Methles cribratellus* (Fairmaire, 1880)		15	1, 6, 7, 13, 16, 17, 18, 22, 23, 27, 31	X	X	X
Derovatellus cf. natalensis Omer-Cooper, 1965	27, 30		16, 17, 19, 22, 23, 27, 29	X	X	X
*Laccophilus canthydroides* Omer-Cooper, 1957			17, 22, 23, 29	X	X	X
*Laccophilus cryptos* Biström, Nilsson & Bergsten, 2015		2	14, 21, 22, 23, 27, 29	X	X	X
*Laccophilus contiro* Guignot, 1952			6, 14, 22, 23, 27, 31	X	X	X
*Laccophilus simplicistriatus* Gschwendtner, 1932	7, 27	7, 15	21, 23, 26, 27, 29, 31	X	X	X

+ Taxa known only from South Africa.

Hydradephagan taxa reported at Lake St Lucia and its immediate surrounds prior to our surveys are listed in Table 4. Of these, some were only identified to genus level. Fifteen of the taxa identified to species in previous studies were also recorded in our surveys. Only three unpublished museum records could be found from extensive searches across the collections of South Africa’s museums. Of these, *Hydaticus bivittatus* Laporte, 1835 was recorded in the current study (Table 3). Four hydradephagan species were collected by the current authors during *ad hoc* collections made in 2008 and 2012 (Table 4), of which *Cybister natalensis* (Wehncke, 1876), *Hydaticus servillianus* Aubé, 1838 and *Rhantaticus congestus* (Klug, 1833) were all recorded during 2013–2015 (Table 3). *Laccophilus lateralis* Sharp, 1882, reported from St Lucia by Day et al. (1954) and Millard and Broekhuysen (1970) is excluded here since Biström et al. (2015) demonstrate that this species is almost certainly restricted to Madagascar, mainland African records representing other, similar species. Similarly *Aethionectes oberthueri* (Régimbart, 1895), reported by the same authors, is excluded from Table 4 as this species is apparently also a Madagascan endemic. It seems more likely that the record refers to *Aethionectes apicalis* (Boheman, 1848), widely reported in southern Africa (Guignot 1961, Omer-Cooper 1966).

**Table 4.** Hydradephagan beetles previously recorded from the Lake St Lucia system and surrounding waterbodies. Literature sources indicated by letters as follows: (**a**) Day et al. (1954); (**b**) Millard and Broekhuysen (1970); (**c**) Vrdoljak (2004); (**d**) Biström and Nilsson (2002); (**e**) Biström et al. (2015). FWS – fresh water streams feeding into South Lake; FWW – fresh water wetlands on the eastern shores of Lake St Lucia; LT – at light, Mission Rock. Also included here are records based on museum material and ad hoc collections undertaken by the authors in 2008 and 2012 (deposited at and listed as UKZN). SANC – South African National Collection of Insects; ISAM – Iziko South African Museum; TMSA – Ditsong National Museum of Natural History; UKZN – University of KwaZulu-Natal; SL – St Lucia (lake body and immediate surrounds); KB – Kosi Bay; DF – Dukuduku forest; DP – Dukandlovu Pan (site 29 in the current study).

Family	Genus	Species	Publication	Years recorded	Location
Gyrinidae	*Dineutus*	Dineutus subspinosus*	(c)	2002/2003	FWW
	*Gyrinus*	Gyrinus natalensis*	(c)	2002/2003	FWW
Noteridae	*Canthydrus*	Canthydrus notula*	(c)	2002/2003	FWW
		*Canthydrus* spp. 1–4	(c)	2002/2003	FWW
	*Hydrocanthus*	Hydrocanthus ferruginicollis*	(a), (b)	1964/1965	FWS
		*Hydrocanthus funebris* Fairmaire, 1869	(c)	2002/2003	FWW
		*Hydrocanthus* spp. 1–2	(c)	2002/2003	FWW
	*Hydrocoptus*	*Hydrocoptus* spp. 1–2	(c)	2002/2003	FWW
	*Synchortus*	*Synchortus* spp. 1–2	(c)	2002/2003	FWW
Dytiscidae	*Rhantus*	*Rhantus* sp.	(c)	2002/2003	FWW
	*Copelatus*	*Copelatus sylvaticus* Guignot, 1952	(c)	2002/2003	FWW
	*Cybister*	*Cybister guignoti* Gschwendtner, 1936	(c)	2002/2003	FWW
		Cybister marginicollis*	(c)	2002/2003	FWW
		Cybister natalensis*	UKZN	2012	FB, DP
		Cybister vulneratus*	(c)	2002/2003	FWW
		*Cybister* sp.	(c)	2002/2003	FWW
	*Aethionectes*	*Aethionectes* sp.	(c)	2002/2003	FWW
	*Rhantaticus*	Rhantaticus congestus*	(c)	2002/2003	FWW
			UKZN	2012	DP
	*Hydaticus*	Hydaticus bivittatus*	SANC	Not specified	SL
		Hydaticus servillanus*	UKZN	2012	FB
		*Hydaticus* sp.	ISAM	1988	KB
	*Pseuduvarus*	Pseuduvarus viticollis*	(c)	2002/2003	FWW
	*Hydrovatus*	*Hydrovatus madagascariensis* Régimbart, 1903	TMSA	1956	DF
	*Herophydrus*	Herophydrus nigrescens*	(d)	1975	LT
		*Herophydrus* spp. 1–3	(c)	2002/2003	FWW
		*Hydrovatus* spp. 1–2	(c)	2002/2003	FWW
	*Hyphydrus*	Hyphydrus cycloides*	(c)	2002/2003	FWW
		*Hydrocanthus grandis* Laporte, 1835	(c)	2002/2003	FWW
		*Hydrocanthus maculatus* Babington, 1841	(c)	2002/2003	FWW
		Hyphydrus signatus*	(a), (b)	1964/1965	FWS
	*Methles*	*Methles* sp.	(c)	2002/2003	FWW
	*Derovatellus*	*Derovatellus* spp. 1–2	(c)	2002/2003	FWW
	*Laccophilus*	*Laccophilus australis* Biström, Nilsson & Bergsten, 2015	(e)	1975	LT
		Laccophilus cryptos*	(e)	1975	LT
		*Laccophilus secundus* Régimbart, 1895	(e)	?	?
		*Laccophilus* spp. 1–5	(c)	2002/2003	FWW

* Also recorded during the dedicated surveys of 2013–2015.

**Figure 4.**
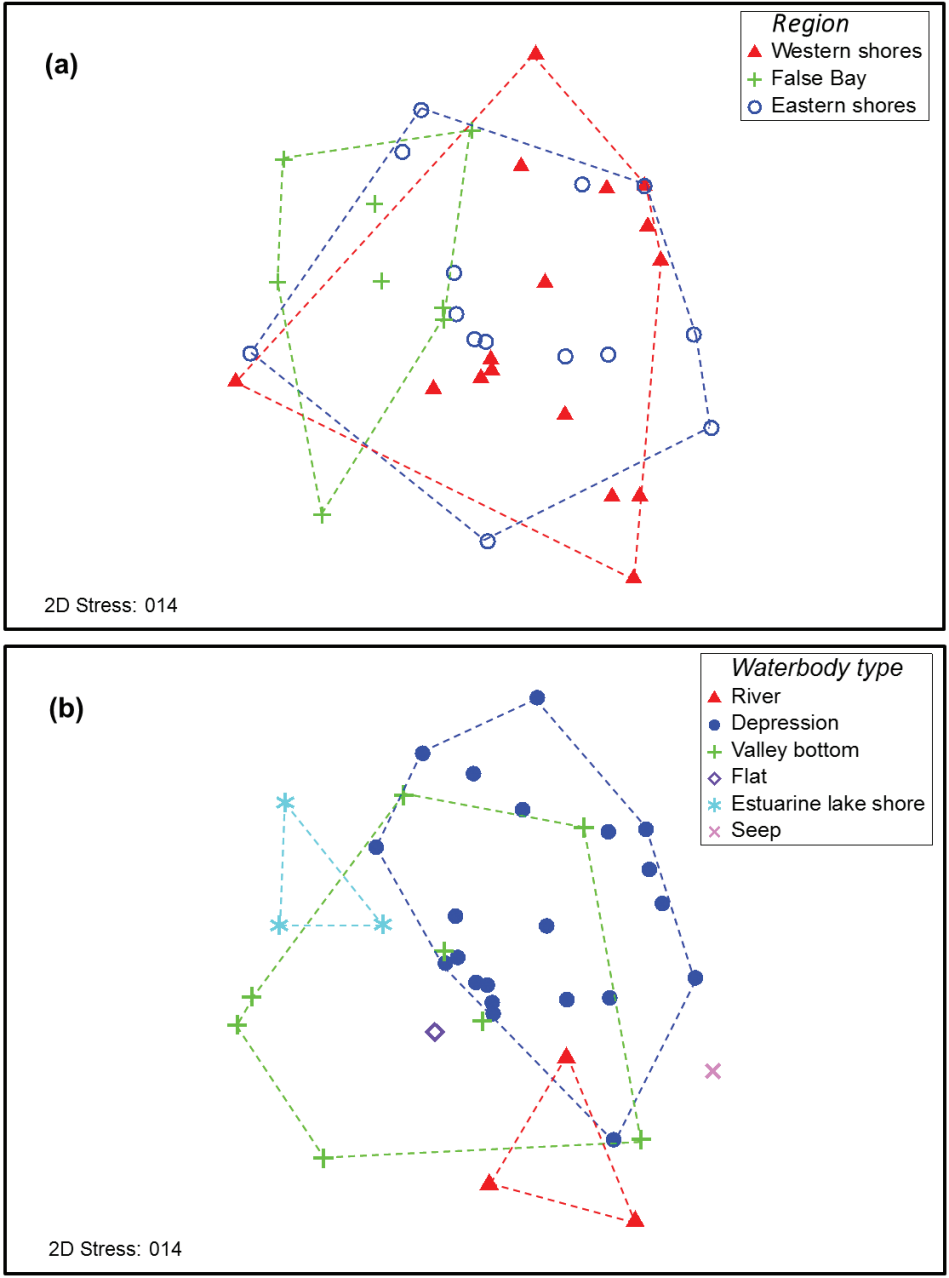
Multidimensional scaling (MDS) plot depicting the similarity of sites sampled in this study in terms of beetle assemblage composition. Symbols on the plot have been coded in terms of **a** region and **b** waterbody type. Convex hulls (dashed lines) have been overlaid on each plot to clarify groupings according to region/waterbody type.

Aquatic beetle assemblage composition of the sites sampled during 2013-2015 differed across both regions and waterbody types (Fig. 4); PERMANOVA tests reveal that these differences were significant (Table 5). In terms of the regions sampled, there appears to be overlap in the MDS plot between the western and eastern shores sites, whilst the False Bay sites appear to be more strongly differentiated (Fig. 4a). This is confirmed by the *post hoc* pairwise comparisons in Table 5a, which demonstrate that the overall significance was driven by differences between False Bay and the other regions. In terms of waterbody type, the different waterbodies formed quite distinct clusters in the MDS, with the exception of valley bottoms, which were scattered widely across the plot (Fig. 4b). The overall significant difference (P < 0.05) between waterbody types is confirmed by the PERMANOVA results in Table 5b, which show that this result is driven by differences between depression wetlands and estuarine lake shores/rivers.

**Table 5.** Non-parametric permutational MANOVA (PERMANOVA) results for models comparing beetle assemblage composition. Assemblage composition at St Lucia was compared across (a) regions, and (b) waterbody types. The multivariate models tested for differences between group centroids in Bray-Curtis dissimilarity space. WS – western shores; FB – False Bay; ES – eastern shores; Dep. – depression wetland; ELS – estuarine lake shore; VB – valley bottom.

(a)						*Post hoc* pairwise comparisons		
Source	df	SS	MS	F	P	Groups	t	P
Region	2	12087	6043.7	1.6119	0.0311*	WS, FB	1.6932	0.0014*
Residual	35	131230	3749.4			WS, ES	0.7968	0.8007
Total	37	143320				FB, ES	1.2882	0.0471*
**(b)**						***Post hoc* pairwise comparisons**		
**Source**	**df**	**SS**	**MS**	**F**	**P**	**Groups**	**t**	**P**
Waterbody type	3	16804	5601.4	1.5174	0.0277*	Dep., ELS	1.3635	0.0368*
Residual	32	118130	3691.6			Dep., River	1.4480	0.0205*
Total	35	134930				Dep., VB	1.1185	0.2522
						ELS, River	1.3239	0.0973
						ELS, VB	0.7162	0.9276
						River, VB	1.1209	0.1860

* Significant P values at α = 0.05.

**Figure 5.**
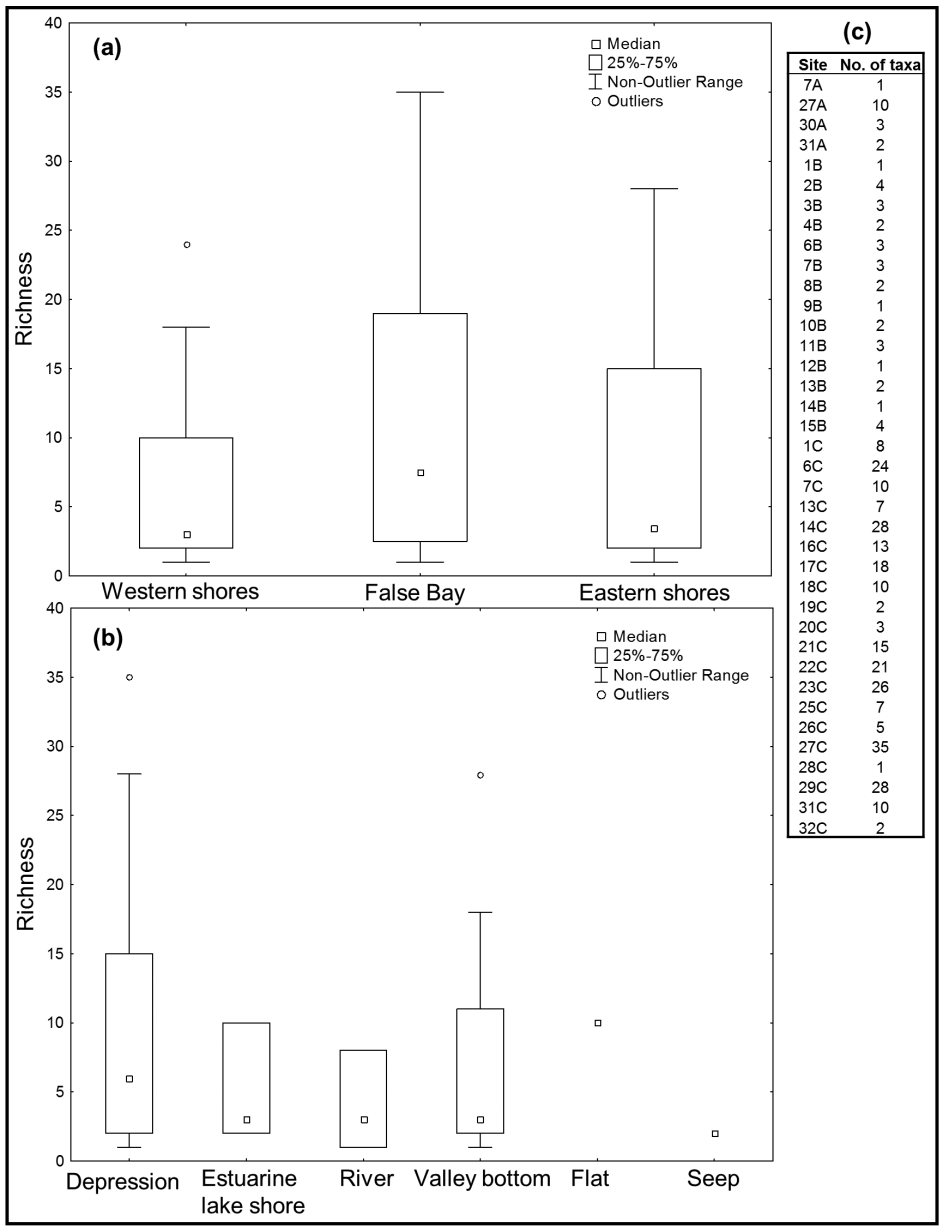
Box-plots comparing the median and spread of species richness (number of hydradephagan taxa per site) among **a** regions and **b** waterbody types at St Lucia during the sampling period 2013–2015. The data representing number of taxa per site are also reported (**c**). Site numbers in (**c**) are coded as A (first survey – November 2013), B (second survey – July 2014) or C (third survey – January/February 2015). Kruskal-Wallis tests indicated that species richness did not vary significantly among regions (KW-H_2, 38_ = 1.0025, p = 0.6058) or waterbody types (KW-H_5, 38_ = 2.273, p = 0.8102).

Non-parametric Kruskal-Wallis tests indicate that species richness did not vary significantly between regions (KW-H_2, 38_ = 1.0025, p = 0.6058) or waterbody types (KW-H_5, 38_ = 2.273, p = 0.8102) at St Lucia. Mean richness across all sites and sampling trips was 8.5±9.3 (±SD) taxa per site, the very high standard deviation indicating that the number of taxa recorded per site was extremely variable. The highest recorded richness for an individual site visit was 35 taxa, collected from site 27 (Mpophomeni pan) at False Bay in January/February 2015. Sites 14 (eastern shores), 23 (eastern shores) and 29 (Dukandlovu Pan, False Bay) all yielded more than 25 taxa during January/February 2015. Yet only a single taxon was recorded from site 7 (eastern shores) in November 2013, sites 1 (western shores), 9 (western shores), 12 (eastern shores) and 14 (eastern shores) in July 2014 and site 28 (False Bay) in January/February 2015 (Fig. 5).

## Discussion

This study reveals that the St Lucia lake system and its associated wetlands support at least 68 species of Hydradephaga. It is currently estimated that ca. 410 species of Hydradephaga occur in southern Africa as a whole (Stals and de Moor 2007), meaning that almost 20% of the known fauna of this biodiverse region occur in the wetlands of the St Lucia system.

The species richness observed at St Lucia is comparable with that recorded in a number of tropical locations worldwide. For example, Bilardo and Rocchi (2011) obtained 51 hydradephagan species in the Monts de Cristal National Park, Gabon, during sampling conducted between 2006 and 2010. Similarly, Apenborn (2013) reported the collection of 122 species of aquatic beetles, representing 10 different families, during an eight-week survey of the Peruvian Amazon near the Panguana Biological Field Station (Hendrich et al. 2015). Of these, around 80 belonged to the Hydradephaga. Lentic sites in northern temperate regions such as the Western Palaearctic can also support relatively diverse hydradephagan assemblages. Foster (1993) summarises records from lithalsa fen complexes in Norfolk, UK, with 65 species being recorded across four sites. Bameul (1994) reported 50 species from the Marais de la Perge, a 3280 ha lithalsa complex close to Bordeaux, France, now decimated by invasive crayfish. Similarly, a total of 29 hydradephagan species were reported from the Lonjsko Polje Nature Park in Croatia (Temunović et al. 2007) and 31 in the Villafáfila shallow lakes of the NW Iberian Meseta, Spain (Régil and Garrido 1993).

Five species of Hydradephaga found during our surveys are apparently new to the fauna of South Africa (Table 3). These are all relatively widespread Afrotropical species, but include the large *Cybister ertli* Zimmermann, 1917. Whilst *Cybister ertli* has been recorded from Swaziland in the past, this species has not previously been reported from South Africa. It is possible that specimens have been confounded with other large black *Cybister* however, as these beetles are not easy to identify without reference to the male genitalia. It is highly likely that the *Peltodytes* found during our surveys is currently undescribed. This does not match any of the known African species in external morphology (van Vondel 2010, D. T. Bilton, *pers. obs.*), but cannot be either positively identified or described at present, in the absence of males.

The hydradephagan fauna of Lake St Lucia is dominated by relatively widespread Afrotropical taxa (see distribution records in Appendix 1), with very few species with restricted distributions, a pattern also seen in other aquatic taxa such as gastropods (Perissinotto et al. 2014) and odonates (Hart et al. 2014). Three of the species recorded in these surveys are currently thought to be endemic to South Africa (Table 3), with only two, *Herophydrus nigrescens* Biström & Nilsson, 2002 and Hydaticus cf. natalensis Guignot, 1951 apparently endemic to KwaZulu-Natal. This dominance of the diverse St Lucia fauna by widespread species fits well into the general principle that hotspots of endemism and species richness are often not coincident (e.g. Stals and de Moor 2007). In many insect taxa, the north-east of South Africa supports relatively high species richness, whilst endemism is instead generally concentrated towards the south-western part of the country, particularly in the Western Cape (Lombard 1995, Bilton 2014b, Bilton et al. 2015). In Table Mountain National Park, for example, a species-rich wetland would typically contain ca. 15–20 species of Hydradephaga (cf. up to 35 at St Lucia). In stark contrast to the fauna of St Lucia, however, around 50% of the species found in a typical Cape Peninsula wetland are endemic to the fynbos biome (D. T. Bilton, *pers. obs.*; Pryke and Samways 2009).

Only three species (*Cybister vulneratus* Klug, 1834, *Hydaticus servillianus* Aubé, 1838 and Derovatellus cf. natalensis Omer-Cooper, 1965) were found in the margins of Lake St Lucia itself, the overwhelming majority of species being associated with small wetlands in the park. False Bay sites supported relatively distinctive beetle assemblages, including species which were not recorded elsewhere, whereas the faunas of the eastern and western shores largely overlapped (Fig. 4 and Table 5). False Bay is considerably more arid than the other two areas and its landscape is dominated by dense dry woodland, in contrast to the moist grassland plains with pockets of dune forest characteristic of the eastern and western shores (Perissinotto et al. 2013). Sites at False Bay were typically heavily shaded by shrub encroachment, whilst those on the eastern and western shores were generally more open and sunlit. Thus the contrasting riparian and terrestrial environments across these broad regions at St Lucia offers a potential explanation for the differentiation of aquatic assemblages in the wetlands of the three areas. This is reflected to some extent in the PCA of physico-chemistry (Fig. 3). Whilst sites from both eastern and western shores are scattered across the plot, the False Bay localities are largely concentrated towards the left, reflecting their relatively low water temperatures.

Although hydradephagan assemblage composition varied significantly between different areas of St Lucia, species richness did not. Similarly, richness did not differ significantly between the different types of waterbodies sampled. Although there was no significant difference in richness among the waterbody types, the sites with the very highest richness were mostly temporary depression wetlands; a pattern which contrasts markedly with other taxa such as molluscs and dragonflies which are most diverse in permanent waters. With the exception of site 29 (a channelled valley bottom wetland) all sites that yielded 20 or more taxa from a single visit were such depression wetlands (sites 6, 14, 22, 23 and 27), highlighting the importance of this habitat for aquatic conservation in the region for the first time.

Due to unprecedented drought conditions in the region and past anthropogenic activities, there have been significant changes in the St Lucia system in recent decades. The estuary mouth closed in 2002 and large-scale desiccation of the lake basins began in 2004 (Perissinotto et al. 2013). At the peak of these events, over 80% of the lake bottom sediments became exposed to the air and hypersaline conditions dominated the lake system, except in the Narrows and at the mouth. Alternation of dry and wet cycles are not new to this estuary, as can be seen for instance in historic records showing the regular occurrence of 4–10 year cycles of either droughts or anomalous wet conditions since at least the early 1900s. Projections of climate change for the next 50-100 years indicate that this situation will persist and possibly intensify, with the most likely scenario being an alternation of extreme droughts followed by floods (Mather et al. 2013). Predaceous water beetle biodiversity at St Lucia is concentrated in small waterbodies, rather than the main lake system, and the consequences of such changes remain unclear, and may depend on the degree to which changes in the lake system cascade through the wider wetland complex. Drought-induced hypersaline conditions and low water levels (including low groundwater levels) are likely to impact wetlands across the region and the main Lake St Lucia itself, and possible impacts on these wetlands should clearly be considered within wider conservation/management plans.

## Conclusions

The St Lucia system, whilst being dominated by relatively widespread Afrotropical water beetles, supports what appears to be one of the most diverse assemblages of Hydradephaga reported in southern Africa. In line with the high species richness and diversity in other groups investigated to date (e.g. odonates, bivalves, gastropods, crabs), the results of the current study further reinforce the biodiversity importance of the iSimangaliso Wetland Park. Much remains to be investigated, particularly with regard to the environmental factors that support this exceptional biodiversity, and how these may be impacted by climatic and other anthropogenic changes in the future.

## Supplementary Material

XML Treatment for Gyrinus natalensis

XML Treatment for Dineutus subspinosus

XML Treatment for Haliplus natalensis

XML Treatment for Peltodytes sp.

XML Treatment for Canthydrus apicicornis

XML Treatment for Canthydrus marshalli

XML Treatment for Canthydrus notula

XML Treatment for Canthydrus quadrivittatus

XML Treatment for Canthydrus sedilloti

XML Treatment for Hydrocanthus grandis

XML Treatment for Hydrocanthus micans

XML Treatment for Hydrocanthus ferruginicollis

XML Treatment for Synchortus imbricatus

XML Treatment for Synchortus desaegeri

XML Treatment for Neohydrocoptus aethiopicus

XML Treatment for Neohydrocoptus angolensis

XML Treatment for Copelatus cf. ejactus

XML Treatment for Copelatus erichsoni

XML Treatment for Copelatus pulchellus

XML Treatment for Cybister gschwendtneri

XML Treatment for Cybister marginicollis

XML Treatment for Cybister natalensis

XML Treatment for Cybister senegalensis

XML Treatment for Cybister tripunctatus africanus

XML Treatment for Cybsiter bimaculatus

XML Treatment for Cybister ertli

XML Treatment for Cybister vicinus

XML Treatment for Cybister vulneratus

XML Treatment for Rhantaticus congestus

XML Treatment for Eretes sticticus

XML Treatment for Hydaticus bivittatus

XML Treatment for Hydaticus exclamationis

XML Treatment for Hydaticus flavolineatus

XML Treatment for Hydaticus cf. natalensis

XML Treatment for Hydaticus matruelis

XML Treatment for Hydaticus servillianus

XML Treatment for Bidessus sharpi

XML Treatment for Clypeodytes meridionalis

XML Treatment for Hydroglyphus farquharensis

XML Treatment for Hydroglyphus lineolatus

XML Treatment for Hydroglyphus zanzibarensis

XML Treatment for Leiodytes hieroglyphicus

XML Treatment for Pseuduvarus viticollis

XML Treatment for Uvarus gschwendtneri

XML Treatment for Hydrovatus acuminatus

XML Treatment for Hydrovatus cribatus

XML Treatment for Hydrovatus dentatus

XML Treatment for Hydrovatus eximius

XML Treatment for Hydrovatus nefandus

XML Treatment for Hydrovatus nigricans

XML Treatment for Hydrovatus oblongipennis

XML Treatment for Hydrovatus obsoletus

XML Treatment for Hydrovatus villiersi

XML Treatment for Hydrovatus visendus

XML Treatment for Herophydrus guineensis

XML Treatment for Herophydrus inquinatus

XML Treatment for Herophydrus nigrescens

XML Treatment for Herophydrus nodieri

XML Treatment for Heterhydrus senegalensis

XML Treatment for Hyphydrus caffer

XML Treatment for Hyphydrus cycloides

XML Treatment for Hyphydrus signatus

XML Treatment for Methles cribratellus

XML Treatment for Derovatellus cf. natalensis

XML Treatment for Laccophilus canthydroides

XML Treatment for Laccophilus cryptos

XML Treatment for Laccophilus contiro

XML Treatment for Laccophilus simplicistriatus

## Figures and Tables

**Figure 6.**
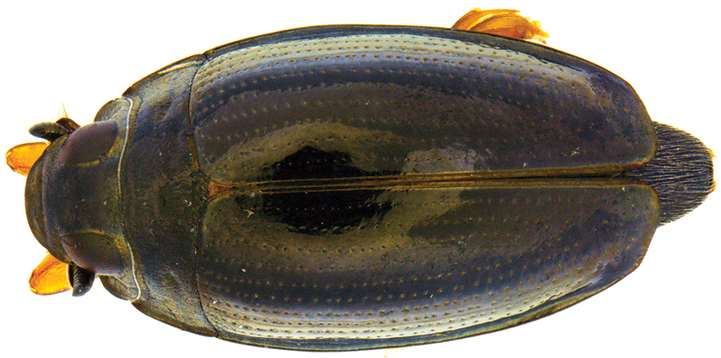
*Gyrinus natalensis* Régimbart, 1892 9.0mm, iSimangaliso Wetland Park, Western Shores (site 4), July 2014 MS Bird leg.

**Figure 7.**
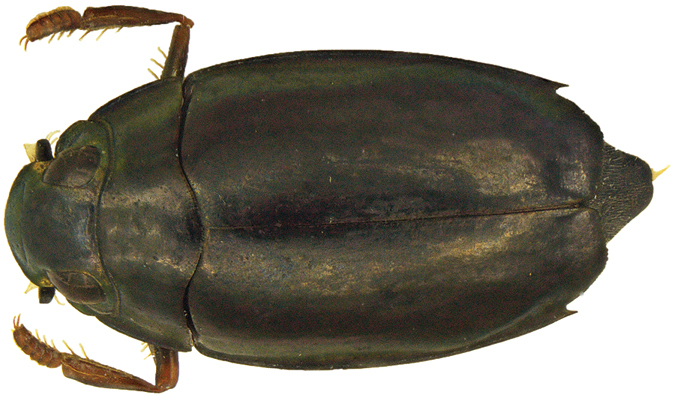
*Dineutus subspinosus* (Klug, 1834) 7.85 mm, iSimangaliso Wetland Park, False Bay (site 29), February 2015 DT Bilton, MS Bird & R Perissinotto leg.

**Figure 8.**
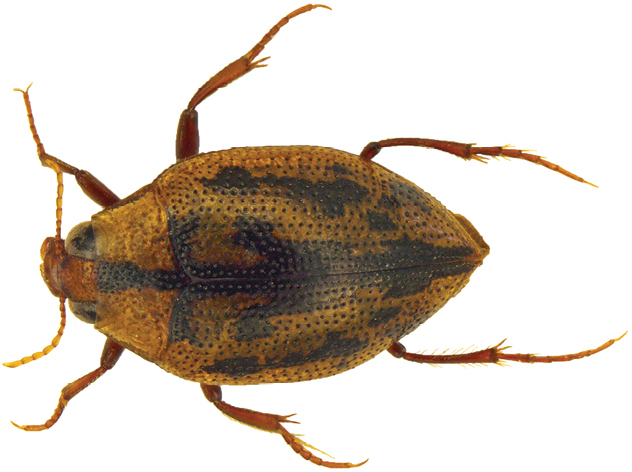
*Haliplus natalenis* Wehncke, 1880 3.72 mm, iSimangaliso Wetland Park, False Bay (site 27), January 2015 DT Bilton, MS Bird & R Perissinotto leg.

**Figure 9.**
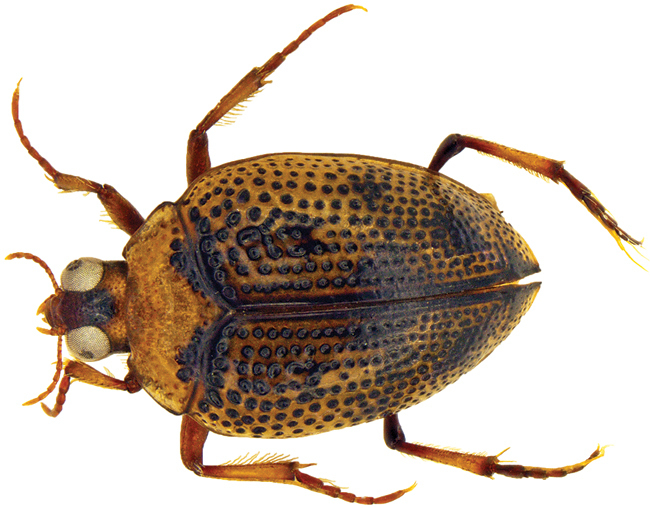
*Peltodytes* sp. 4.10 mm, iSimangaliso Wetland Park, Western Shores (site 6), February 2015 DT Bilton, MS Bird & R Perissinotto leg.

**Figure 10.**
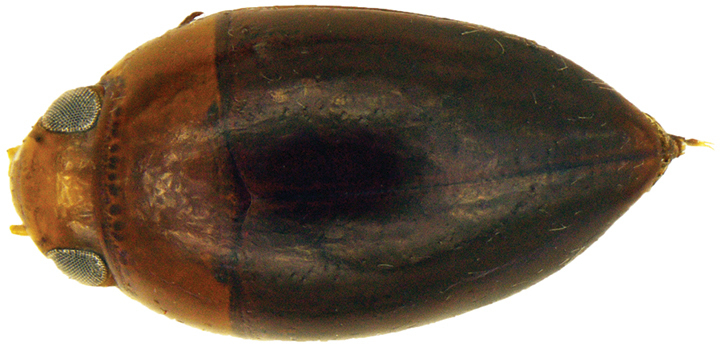
*Canthydrus apicicornis* Régimbart, 1895 2.47 mm, iSimangaliso Wetland Park, Eastern Shores (site 14), February 2015 DT Bilton, MS Bird & R Perissinotto leg.

**Figure 11.**
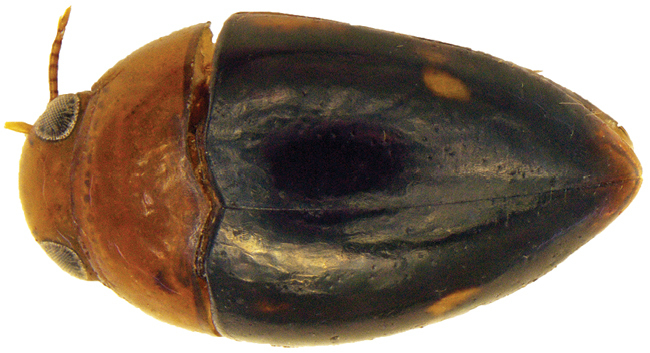
*Canthydrus marshalli* Balfour-Borwne, 1939 2.28 mm, iSimangaliso Wetland Park, Western Shores (site 6), February 2015 DT Bilton, MS Bird & R Perissinotto leg.

**Figure 12.**
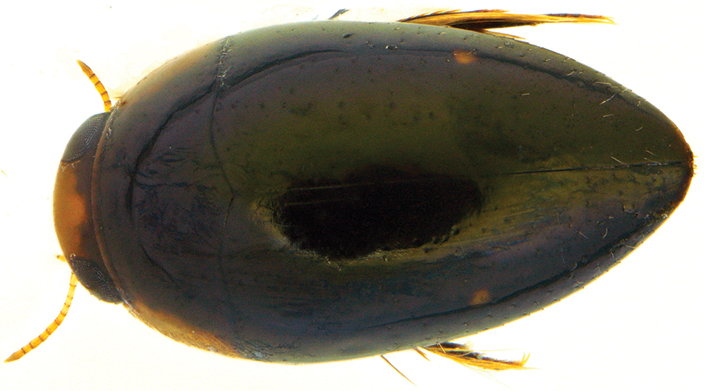
*Canthydrus notula* (Erichson, 1843) 3.28 mm, iSimangaliso Wetland Park, Eastern Shores (site 14), February 2015 DT Bilton, MS Bird & R Perissinotto leg.

**Figure 13.**
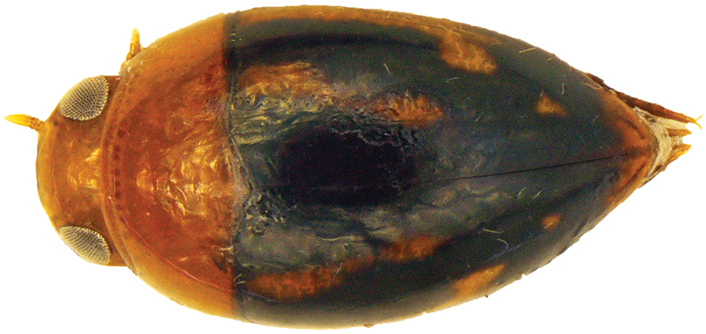
*Canthydrus quadrivittatus* (Boheman, 1848) 2.36 mm, iSimangaliso Wetland Park, Eastern Shores (site 14), February 2015 DT Bilton, MS Bird & R Perissinotto leg.

**Figure 14.**
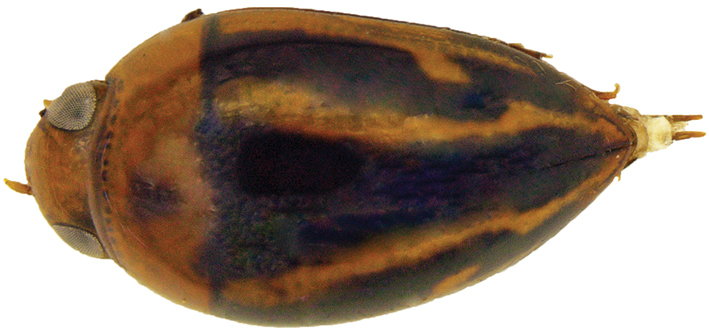
*Canthydrus sedilloti* Régimbart, 1865 2.85 mm, iSimangaliso Wetland Park, Eastern Shores (site 14), February 2015 DT Bilton, MS Bird & R Perissinotto leg.

**Figure 15.**
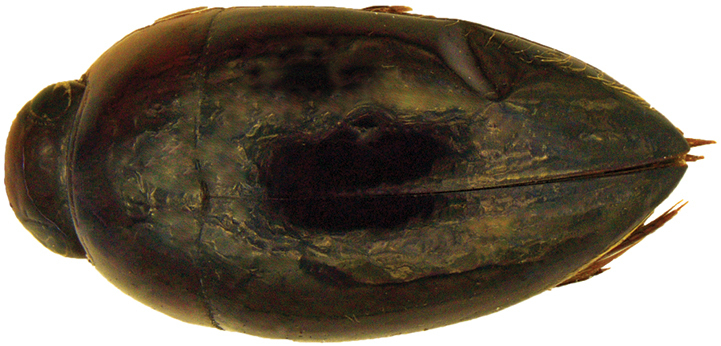
*Hydrocanthus grandis* (Laporte, 1835) 7.40 mm, iSimangaliso Wetland Park, Eastern Shores (site 14), February 2015 DT Bilton, MS Bird, R Perissinotto leg.

**Figure 16.**
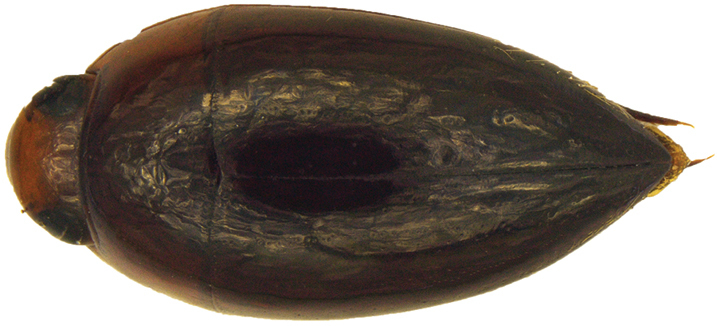
*Hydrocanthus micans* Wehncke, 1883 5.59 mm, iSimangaliso Wetland Park, Eastern Shores (site 14), February 2015 DT Bilton, MS Bird, R Perissinotto leg.

**Figure 17.**
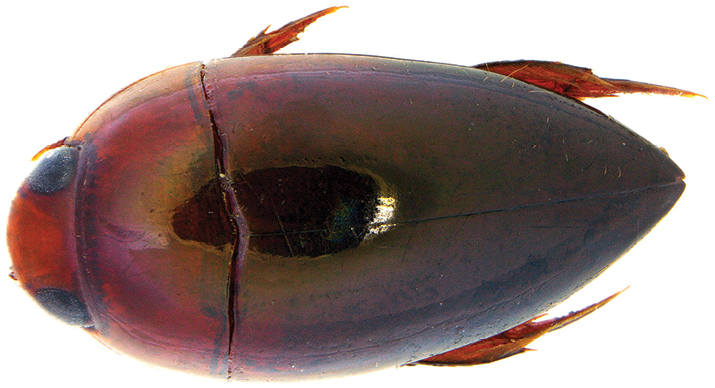
*Hydrocanthus ferruginicollis* Régimbart, 1865 5.00 mm, iSimangaliso Wetland Park, False Bay (site 27), November 2013 MS Bird & R Perissinotto leg.

**Figure 18.**
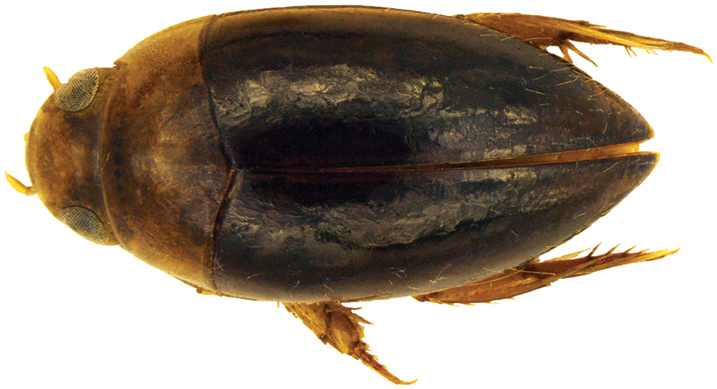
*Synchortus imbricatus* (Klug, 1853) 3.47 mm, iSimangaliso Wetland Park, Eastern Shores (site 14), February 2015 DT Bilton, MS Bird & R Perissinotto leg.

**Figure 19.**
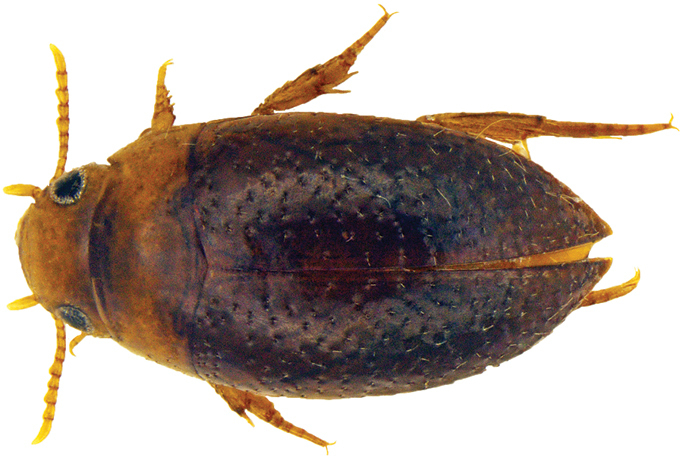
*Synchrotus desaegeri* Gschwendtner, 1935 2.94 mm, iSimangaliso Wetland Park, Eastern Shores (site 23), February 2015 DT Bilton, MS Bird, R Perissinotto leg.

**Figure 20.**
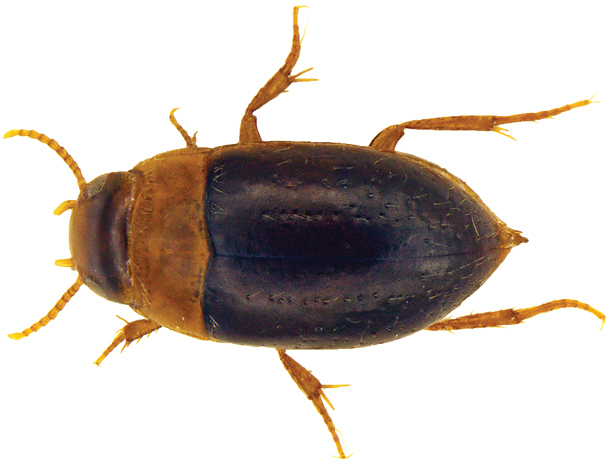
*Neohydrocoptus aethiopicus* (Balfour-Browne, 1961) 2.47 mm, iSimangaliso Wetland Park, Western Shores (site 1), February 2015 DT Bilton, MS Bird, R Perissinotto leg.

**Figure 21.**
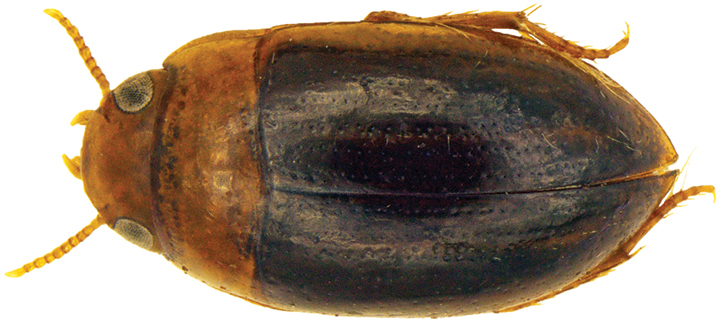
*Neohydrocoptus angolensis* (Peschet, 1925) 2.94 mm, iSimangaliso Wetland Park, Western Shores (site 6), February 2015 DT Bilton, MS Bird & R Perissinotto leg.

**Figure 22.**
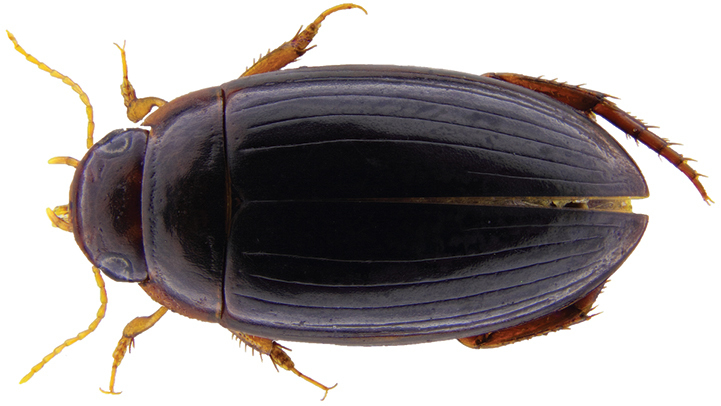
*Copelatus ejactus* Omer-Cooper, 1965 6.75 mm, iSimangaliso Wetland Park, Eastern Shores (site 22), February 2015 DT Bilton, MS Bird & R Perissinotto leg.

**Figure 23.**
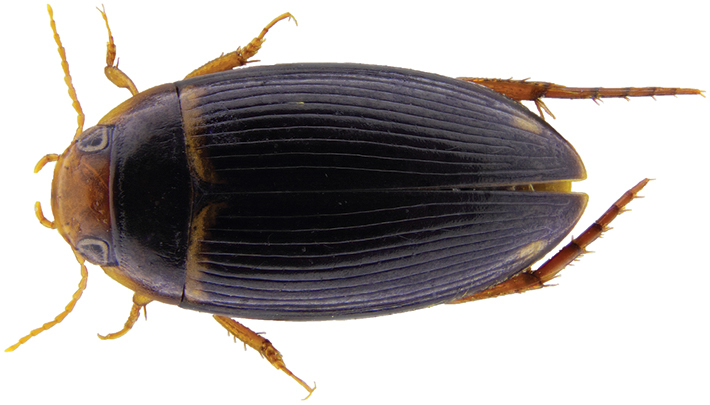
*Copelatus erichsoni* Guérin-Méneville, 1849 5.6 mm, iSimangaliso Wetland Park, Eastern Shores (site 20), February 2015 DT Bilton, MS Bird & R Perissinotto leg.

**Figure 24.**
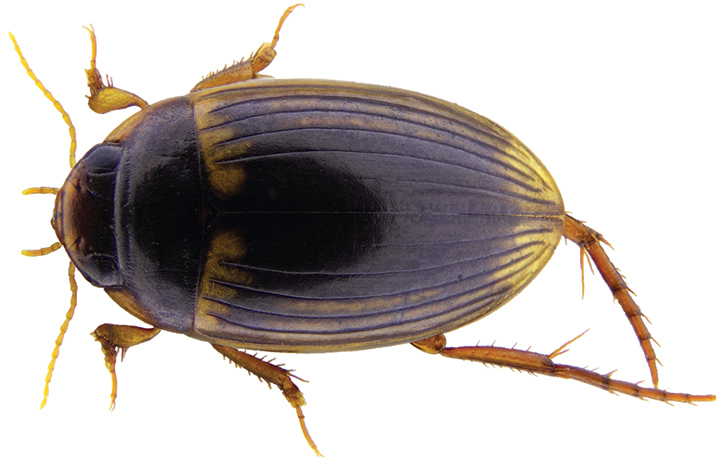
*Copelatus pulchellus* (Klug, 1834) 6.1 mm, iSimangaliso Wetland Park, False Bay (site 26), February 2015 DT Bilton, MS Bird & R Perissinotto leg.

**Figure 25.**
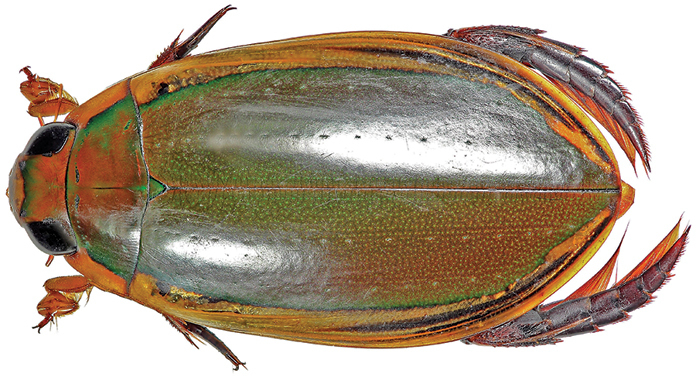
*Cybister gschwendtneri* Guignot, 1935 28.7 mm, iSimangaliso Wetland Park, False Bay (site 27), January 2015 DT Bilton, MS Bird & R Perissinotto leg.

**Figure 26.**
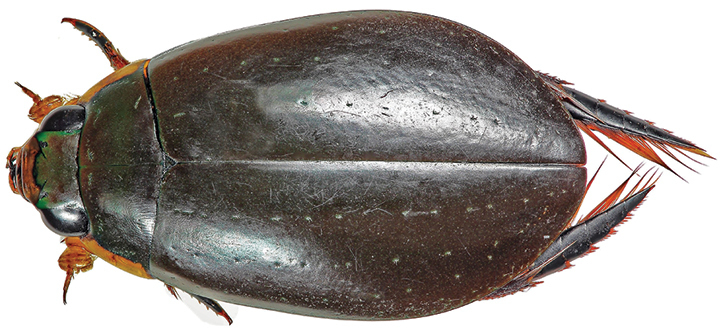
*Cybister marginicollis* Boheman, 1848 15.9 mm, iSimangaliso Wetland Park, False Bay (site 27), January 2015 DT Bilton, MS Bird & R Perissinotto leg.

**Figure 27.**
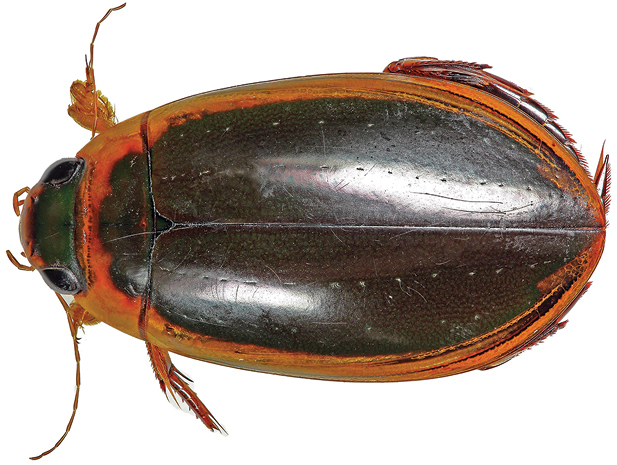
*Cybister natalensis* (Wehncke, 1876) 28.6 mm, iSimangaliso Wetland Park, False Bay (site 29), February 2015 DT Bilton, MS Bird & R Perissinotto leg.

**Figure 28.**
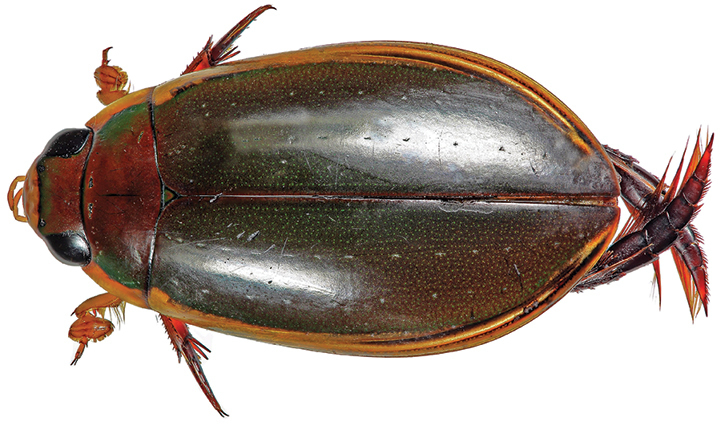
*Cybister senegalensis* Aubé, 1838 20.3 mm, iSimangaliso Wetland Park, Western Shores (site 6), February 2015 DT Bilton, MS Bird & R Perissinotto leg.

**Figure 29.**
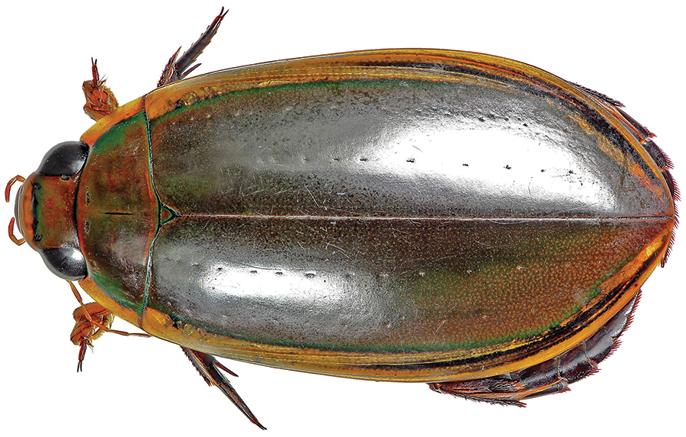
*Cybister tripunctatus africanus* Laporte, 1835 28.4 mm, iSimangaliso Wetland Park, Eastern Shores (site 21), February 2015 DT Bilton, MS Bird & R Perissinotto leg.

**Figure 30.**
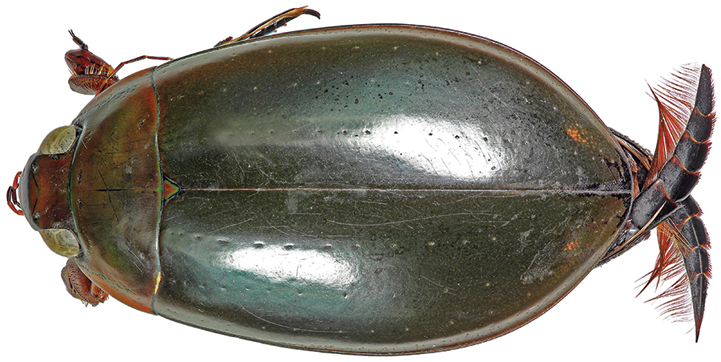
*Cybister bimaculatus* Aubé, 1838 41.4 mm, iSimangaliso Wetland Park, Eastern Shores (21), February 2015 DT Bilton, MS Bird & R Perissinotto leg.

**Figure 31.**
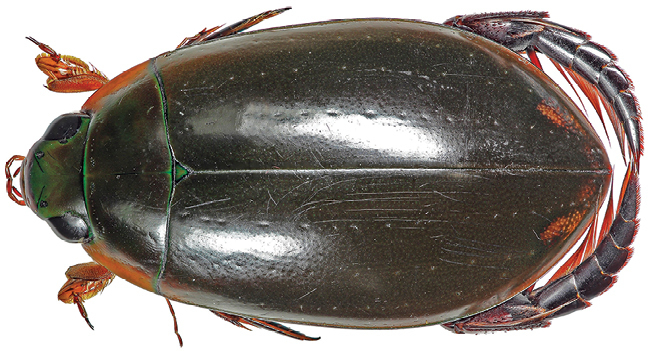
*Cybister ertli* Zimmermann, 1917 38.7 mm, iSimangaliso Wetland Park, Eastern Shores (site 14), February 2015 DT Bilton, MS Bird & R Perissinotto leg.

**Figure 32.**
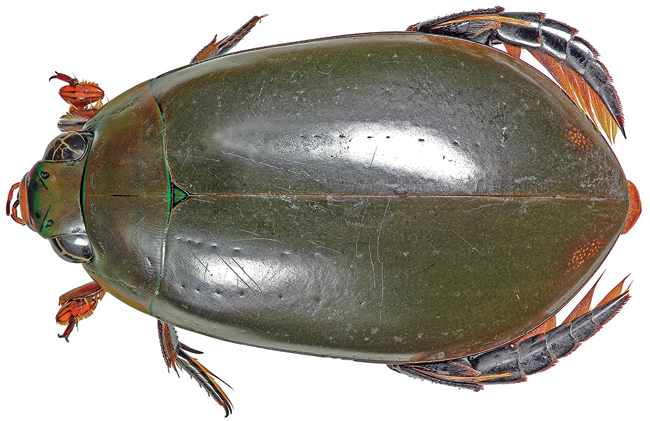
*Cybister vicinus* Zimmermann, 1917 36.7 mm, iSimangaliso Wetland Park, Eastern Shores (site 21), February 2015 DT Bilton, MS Bird & R Perissinotto leg.

**Figure 33.**
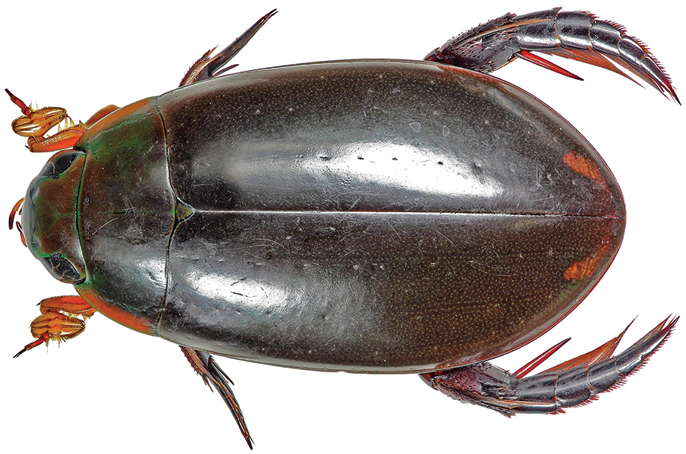
*Cybister vulneratus* Klug, 1834 23.6 mm, iSimangaliso Wetland Park, False Bay (site 27), January 2015 DT Bilton, MS Bird & R Perissinotto leg.

**Figure 34.**
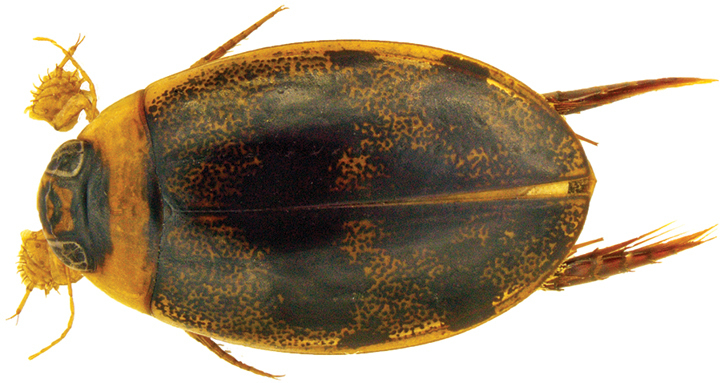
*Rhantaticus congestus* (Klug, 1833) 9.96 mm, iSimangaliso Wetland Park, Eastern Shores (site 21), February 2015 DT Bilton, MS Bird & R Perissinotto leg.

**Figure 35.**
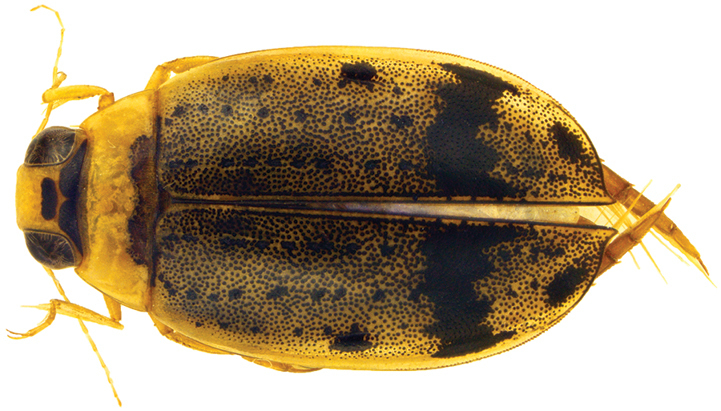
*Eretes sticticus* (Linnaeus, 1767) 12.71 mm, iSimangaliso Wetland Park, Eastern Shores (site 21), February 2015 DT Bilton, MS Bird & R Perissinotto leg.

**Figure 36.**
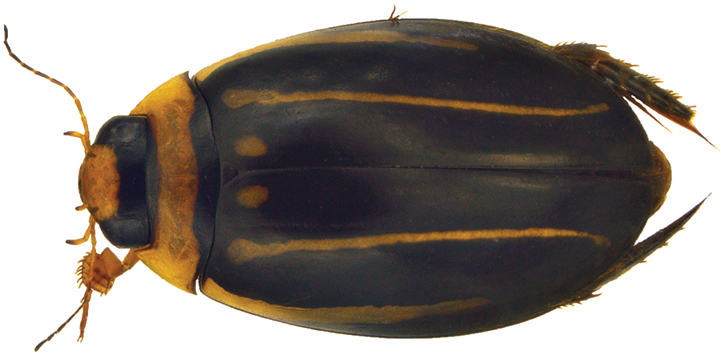
*Hydaticus bivittatus* Laporte, 1835 13.41 mm, iSimangaliso Wetland Park, False Bay (site 27), January 2015 DT Bilton, MS Bird & R Perissinotto leg.

**Figure 37.**
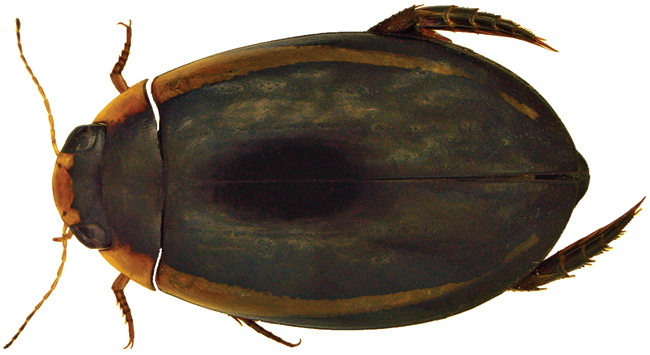
*Hydaticus exclamationis* Aubé, 1838 13.71 mm, iSimangaliso Wetland Park, False Bay (site 27), January 2015 DT Bilton, M Bird & R Perissinotto leg.

**Figure 38.**
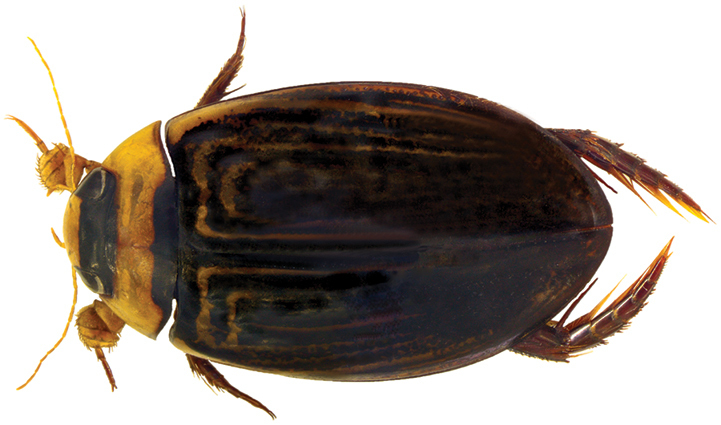
*Hydaticus flavolineatus* Boheman, 1848 16.47 mm, iSimangaliso Wetland Park False Bay (site 27), January 2015 DT Bilton, MS Bird & R Perissinotto leg.

**Figure 39.**
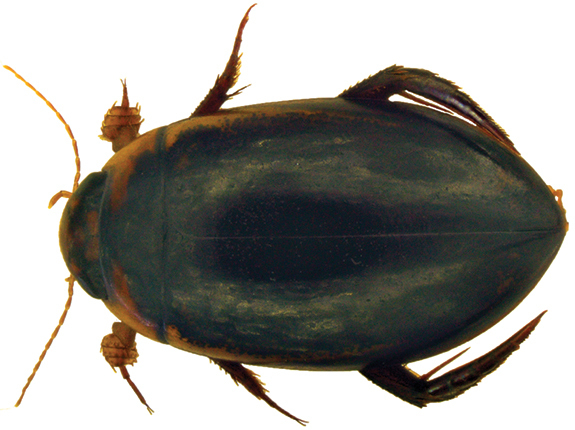
Hydaticus cf. natalensis Guignot, 1951 11.39 mm, iSimangaliso Wetland Park, False Bay (site 27), January 2015 DT Bilton, MS Bird & R Perissinotto leg.

**Figure 40.**
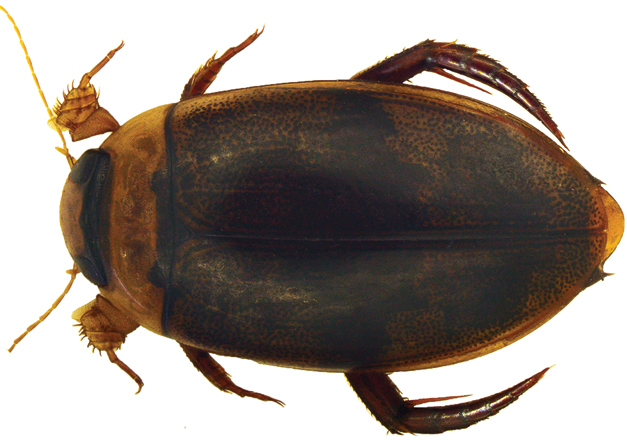
*Hydaticus matruelis* Clark, 1864 11.38 mm, iSimangaliso Wetland Park, False Bay (site 27), January 2015 DT Bilton, MS Bird & R Perissinotto leg.

**Figure 41.**
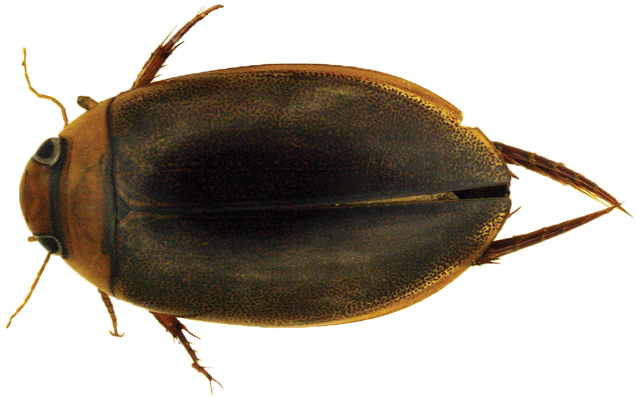
*Hydaticus servillianus* Aubé, 1838 11.23 mm, iSimangaliso Wetland Park, Eastern Shores (site 14), February 2015 DT Bilton, MS Bird & R Perissinotto leg.

**Figure 42.**
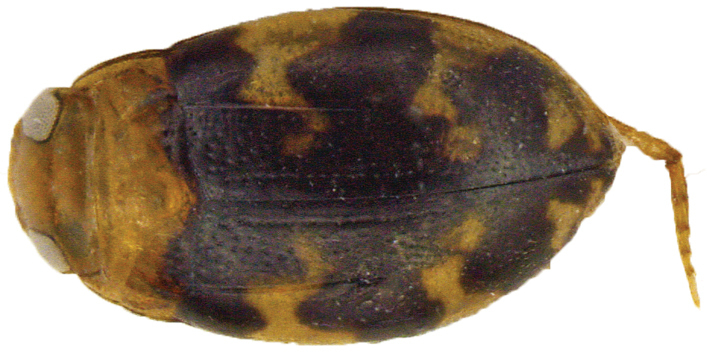
*Bidessus sharpi* Régimbart, 1895 1.67 mm, iSimangaliso Wetland Park, False Bay (site 27), January 2015 DT Bilton, MS Bird & R Perissinotto leg.

**Figure 43.**
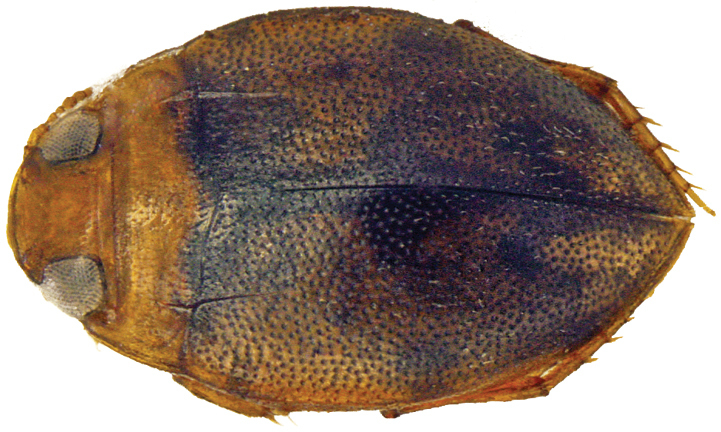
*Clypeodytes meridionalis* Régimbart, 1895 1.93 mm, iSimangaliso Wetland Park, Eastern Shores (site 14), February 2015 DT Bilton, MS Bird & R Perissinotto leg.

**Figure 44.**
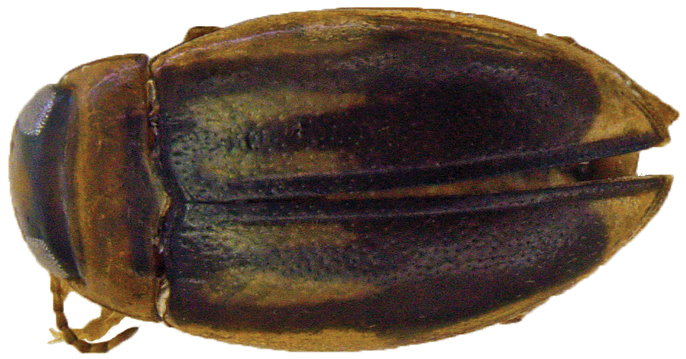
*Hydroglyphus farquharensis* (Scott, 1912) 2.15 mm, iSimangaliso Wetland Park, False Bay (site 27), January 2015 DT Bilton, MS Bird & R Perissinotto leg.

**Figure 45.**
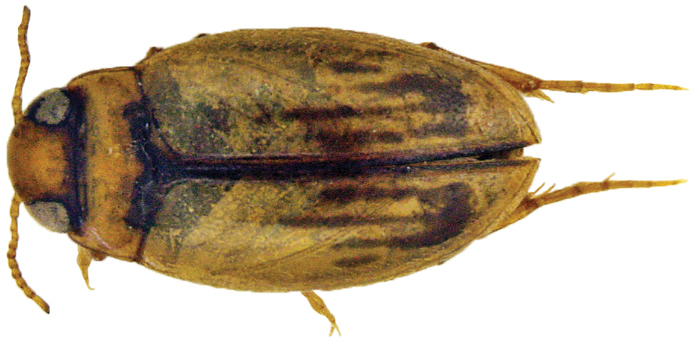
*Hydroglyphus lineolatus* (Boheman, 1848) 2.82 mm, iSimangaliso Wetland Park, Eastern Shores (site 21), February 2015 DT Bilton, MS Bird & R Perissinotto leg.

**Figure 46.**
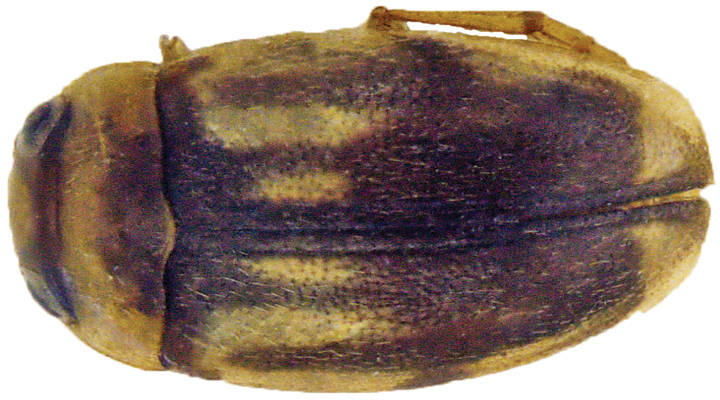
*Hydroglyphus zanzibarensis* (Régimbart, 1906) 1.89 mm, iSimangaliso Wetland Park, False Bay (site 27), January 2015 DT Bilton, MS Bird & R Perissinotto leg.

**Figure 47.**
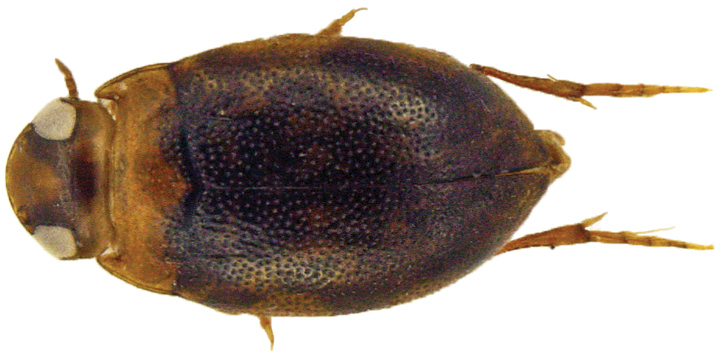
*Leiodytes hieroglyphicus* (Régimbart, 1894) 2.04 mm, iSimangaliso Wetland Park, Western Shores (site 7), February 2015 DT Bilton, MS Bird & R Perissinotto leg.

**Figure 48.**
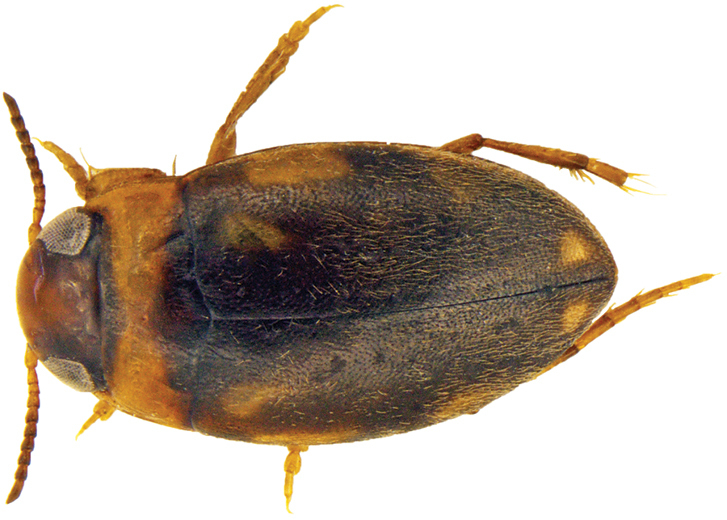
*Pseuduvarus viticollis* (Boheman, 1848) 2.05 mm, iSimangaliso Wetland Park, Catalina Bay (site 32 - seepages over peat), February 2015 DT Bilton, MS Bird & R Perissinotto leg.

**Figure 49.**
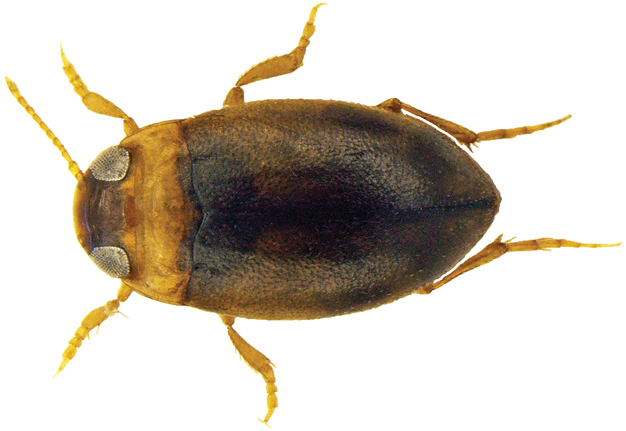
*Uvarus gschwendtneri* (Guignot, 1942) 1.62 mm, iSimangaliso Wetland Park, False Bay (site 27), January 2015 DT Bilton, MS Bird & R Perissinotto leg.

**Figure 50.**
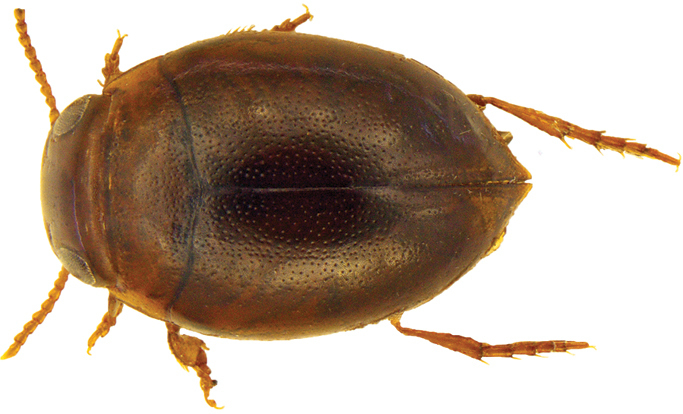
*Hydrovatus acuminatus* Motschulsky, 1859 2.53 mm, iSimangaliso Wetland Park, Eastern Shores (site 14), February 2015 DT Bilton, MS Bird & R Perissinotto leg.

**Figure 51.**
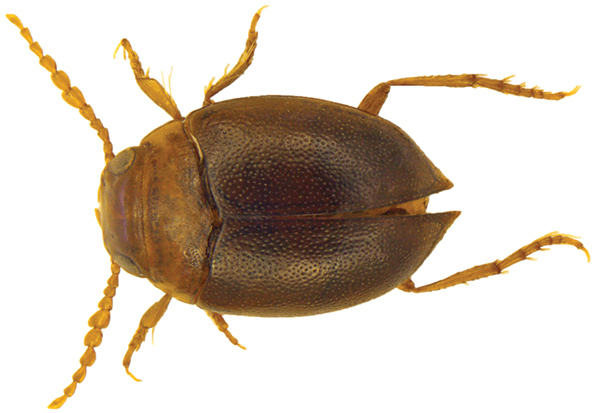
*Hydrovatus cribatus* Sharp, 1882 2.70 mm, iSimangaliso Wetland Park, Eastern Shores (site 14), February 2015 DT Bilton, MS Bird & R Perissinotto leg.

**Figure 52.**
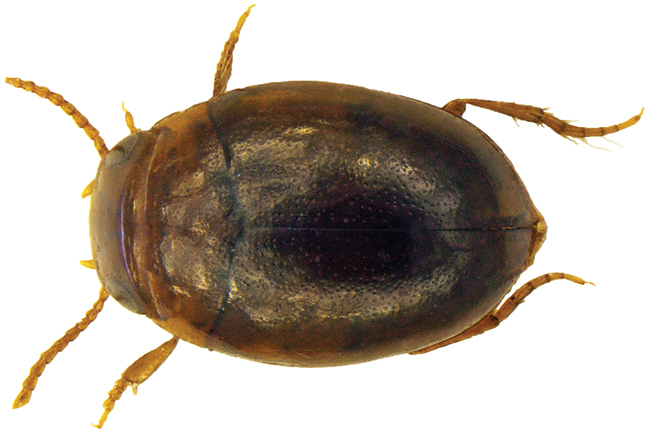
*Hydrovatus dentatus* Bilardo & Rocchi, 1990 2.45 mm, iSimangaliso Wetland Park, Eastern Shores (site 14), February 2015 DT Bilton, MS Bird & R Perissinotto leg.

**Figure 53.**
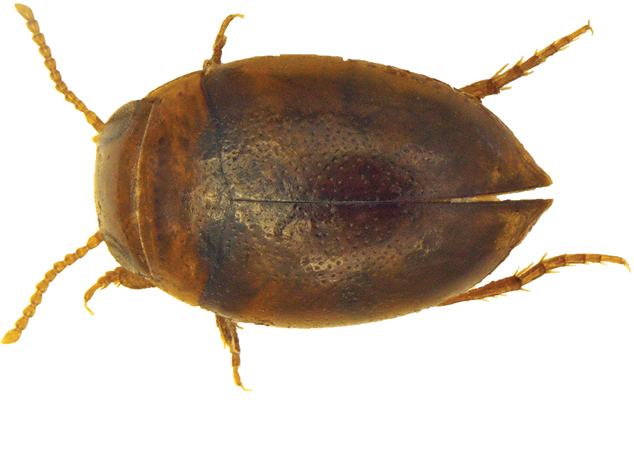
*Hydrovatus eximius* Biström, 1997 2.24 mm, iSimangaliso Wetland Park, False Bay (site 29), February 2015 DT Bilton, MS Bird & R Perissinotto leg.

**Figure 54.**
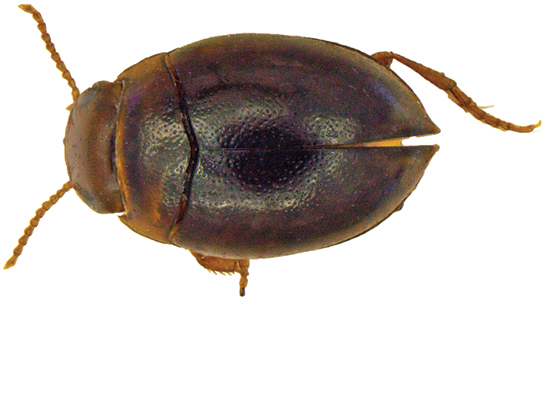
*Hydrovatus nefandus* Omer-Cooper, 1957 2.53 mm, iSimangaliso Wetland Park, False Bay (site 27), January 2015 DT Bilton, MS Bird & R Perissinotto leg.

**Figure 55.**
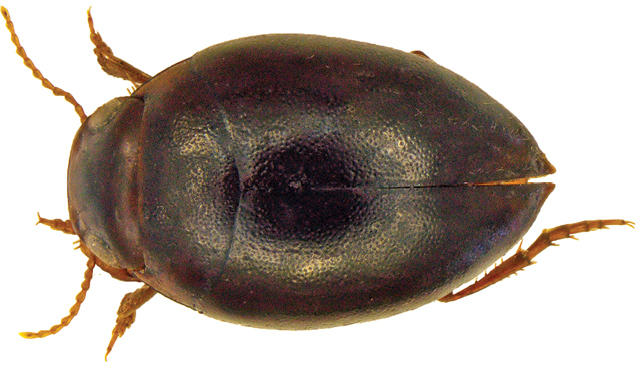
*Hydrovatus nigricans* Sharp, 1882 4.10 mm, iSimangaliso Wetland Park, Eastern Shores (site 23), February 2015 DT Bilton, MS Bird & R Perissinotto leg.

**Figure 56.**
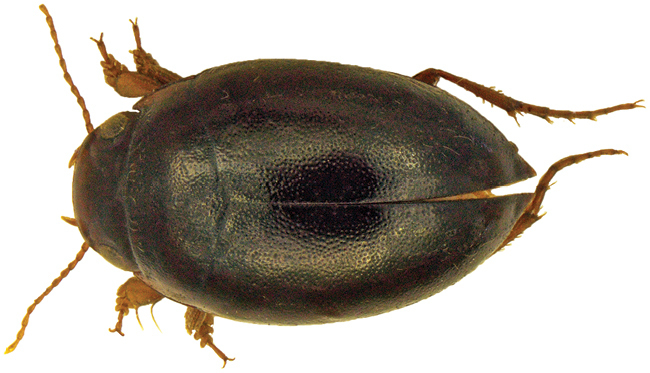
*Hydrovatus oblongipennis* Régimbart, 1895 5.10 mm, iSimangaliso Wetland Park, Eastern Shores (site 23), February 2015 DT Bilton, MS Bird & R Perissinotto leg.

**Figure 57.**
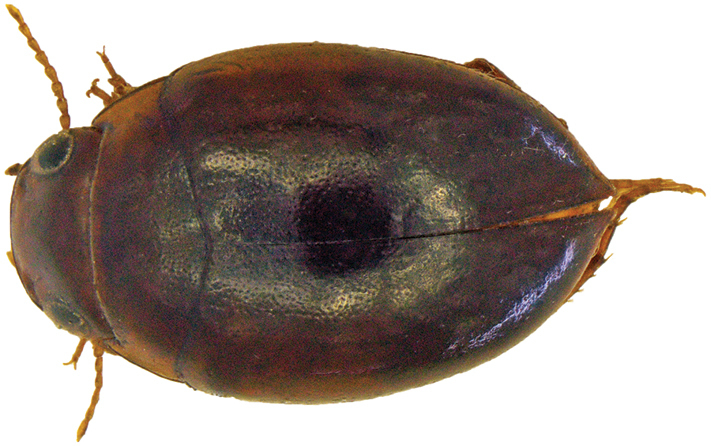
*Hydrovatus obsoletus* Peschet, 1922 3.89 mm, iSimangaliso Wetland Park, Eastern Shores (site 23), February 2015 DT Bilton, MS Bird & R Perissinotto leg.

**Figure 58.**
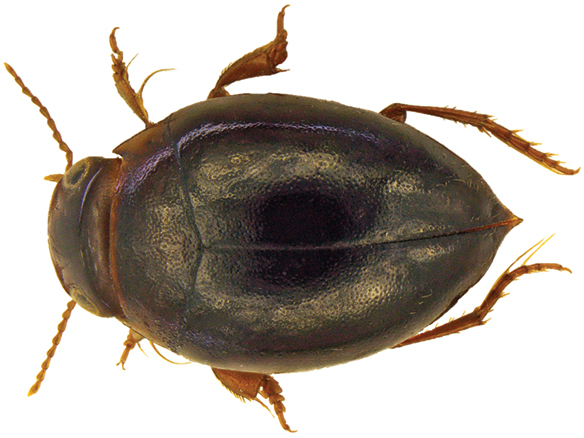
*Hydrovatus villiersi* Guignot, 1955 4.23 mm, iSimangaliso Wetland Park, Eastern Shores (site 14), February 2015 DT Bilton, MS Bird & R Perissinotto leg.

**Figure 59.**
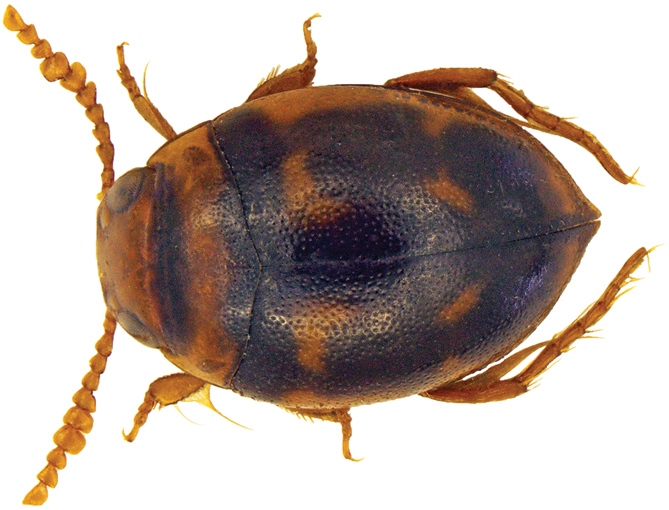
*Hydrovatus visendus* Biström, 1997 3.10 mm, iSimangaliso Wetland Park, Western Shores (site 6), February 2015 DT Bilton, MS Bird & R Perissinotto leg.

**Figure 60.**
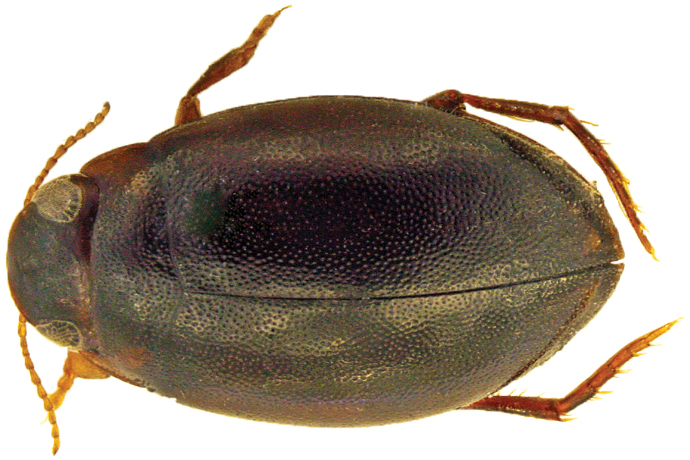
*Herophydrus guineensis* (Aubé, 1838) 4.45 mm, iSimangaliso Wetland Park, False Bay (site 27), January 2015 DT Bilton, MS Bird & R Perissinotto leg.

**Figure 61.**
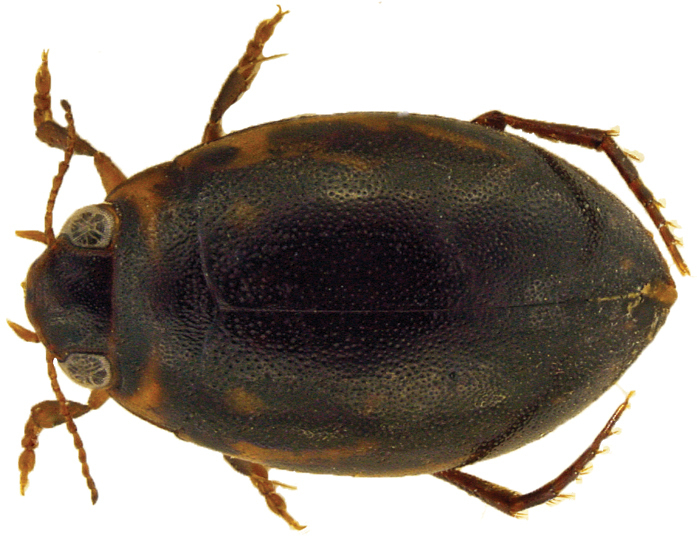
*Herphydrus inquinatus* (Boheman, 1848) 5.27 mm, iSimangaliso Wetland Park, False Bay (site 27), January 2015 DT Bilton, MS Bird & R Perissinotto leg.

**Figure 62.**
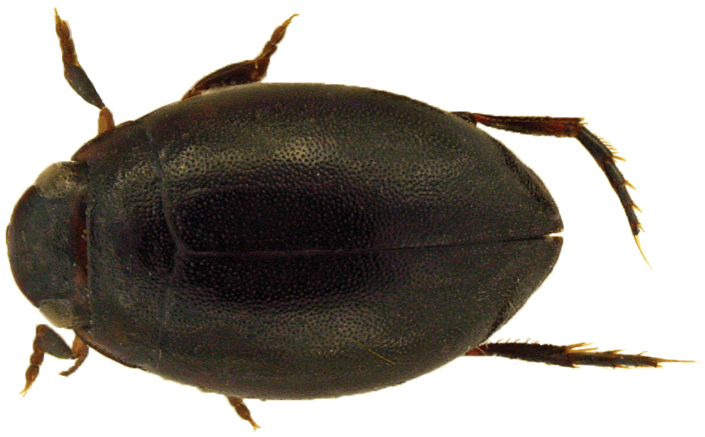
*Herophydrus nigrescens* Biström & Nilsson, 2002 5.31 mm, iSimangaliso Wetland Park, False Bay (site 27), January 2015 DT Bilton, MS Bird & R Perissinotto leg.

**Figure 63.**
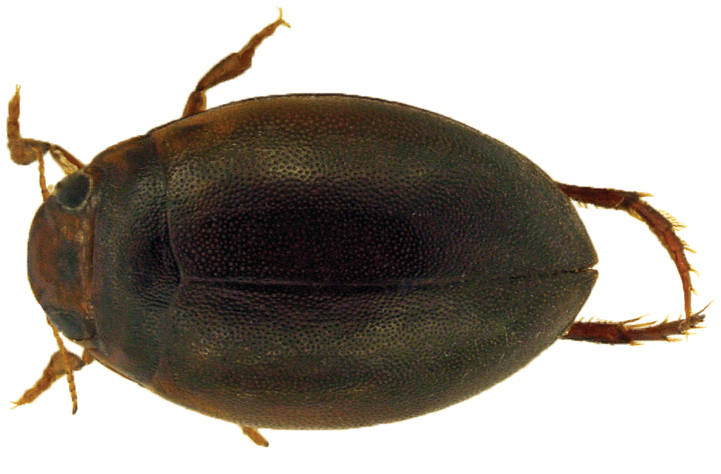
*Herophydrus nodieri* (Régimbart, 1895) 5.66 mm, iSimangaliso Wetland Park, False Bay (site 27), January 2015 DT Bilton, MS Bird & R Perissinotto leg.

**Figure 64.**
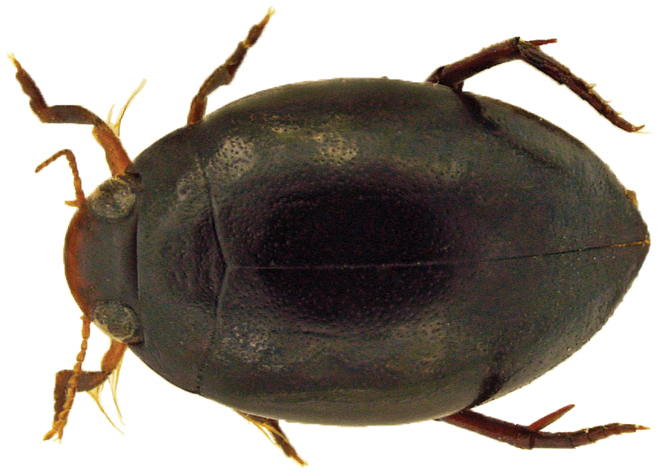
*Heterhydrus senegalensis* (Laporte, 1835) 5.56 mm, iSimangaliso Wetland Park, Eastern Shores (site 14), February 2015 DT Bilton, MS Bird & R Perissinotto leg.

**Figure 65.**
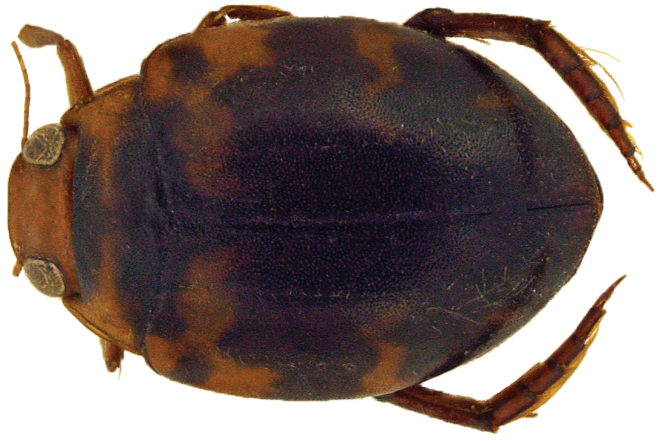
*Hyphydrus caffer* Boheman, 1848 6.06 mm, iSimangaliso Wetland Park, Eastern Shores (site 14), February 2015 DT Bilton, MS Bird & R Perissinotto leg.

**Figure 66.**
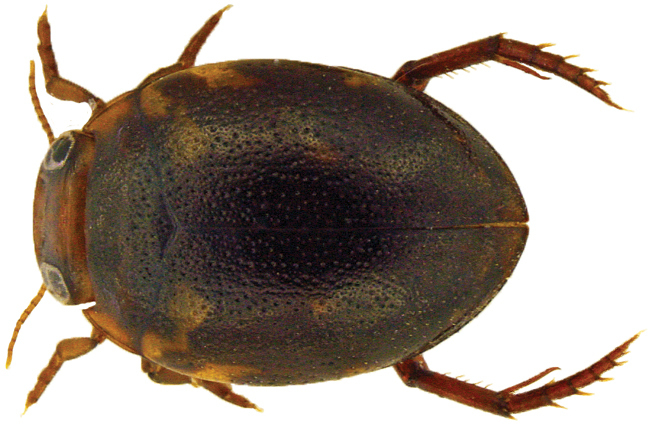
*Hyphydrus cycloides* Régimbart, 1889 3.35 mm, iSimangaliso Wetland Park, Eastern Shores (site 14), February 2015 DT Bilton, MS Bird & R Perissinotto leg.

**Figure 67.**
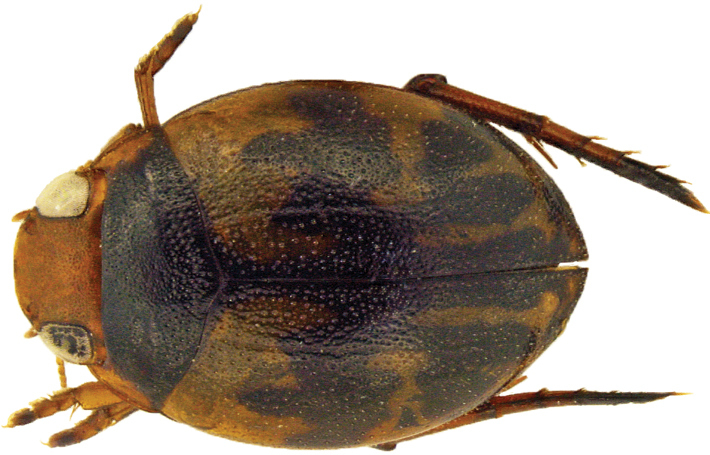
*Hyphydrus signatus* Sharp, 1882 4.52 mm, iSimangaliso Wetland Park, False Bay (site 27), January 2015 DT Bilton, MS Bird & R Perissinotto leg.

**Figure 68.**
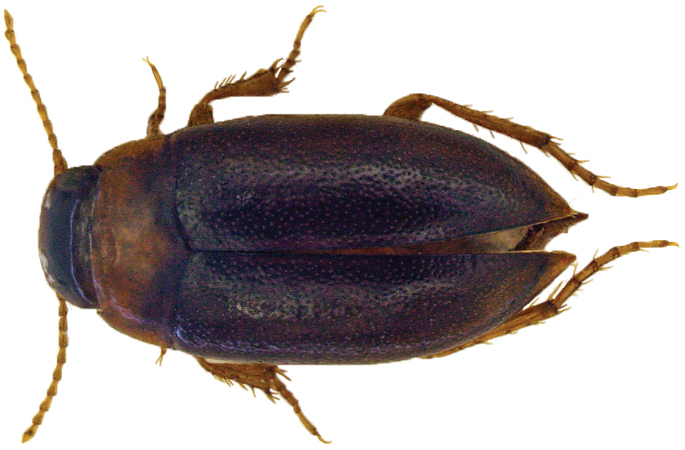
*Methles cribratellus* (Fairmaire, 1880) 3.87 mm, iSimangaliso Wetland Park, False Bay (site 27), January 2015 DT Bilton, MS Bird & R Perissinotto leg.

**Figure 69.**
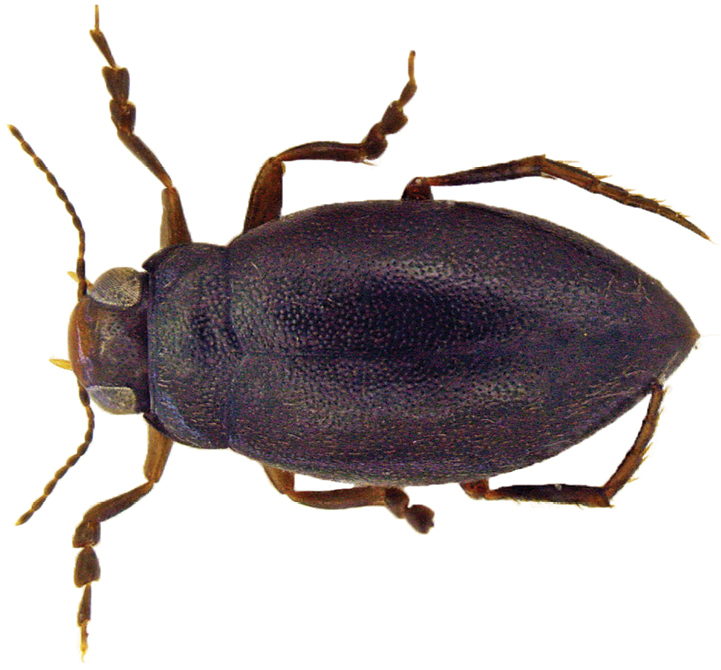
Derovatellus cf. natalensis Omer-Cooper, 1965 3.91 mm, iSimangaliso Wetland Park, False Bay (site 27), January 2015 DT Bilton, MS Bird & R Perissinotto leg.

**Figure 70.**
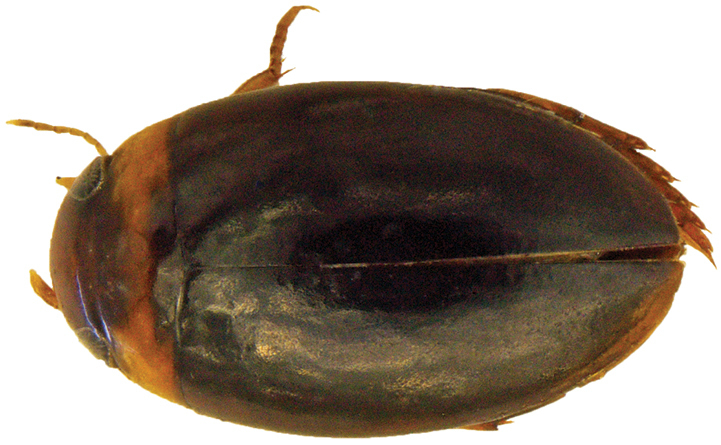
*Laccophilus canthydroides* Omer-Cooper, 1957 3.24 mm, iSimangaliso Wetland Park, Eastern Shores (site 23), February 2015 DT Bilton, MS Bird & R Perissinotto leg.

**Figure 71.**
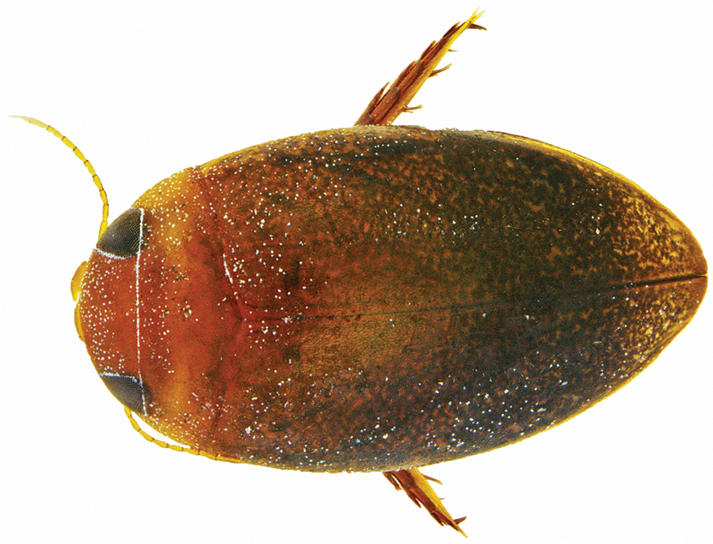
*Laccophilus cryptos* Biström, Nilsson & Bergsten, 2015 2.5 mm, iSimangaliso Wetland Park, Western Shores (site 2), July 2014 MS Bird leg.

**Figure 72.**
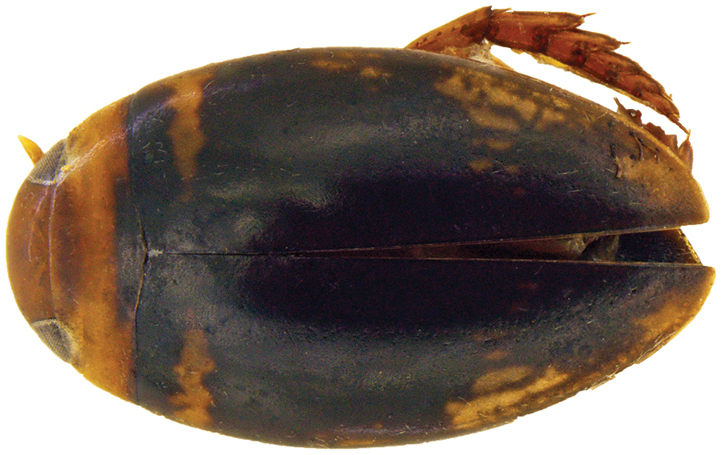
*Laccophilus contiro* Guignot, 1952 3.30 mm, iSimangaliso Wetland Park, Eastern Shores (site 14), February 2015 DT Bilton, MS Bird & R Perissinotto leg.

**Figure 73.**
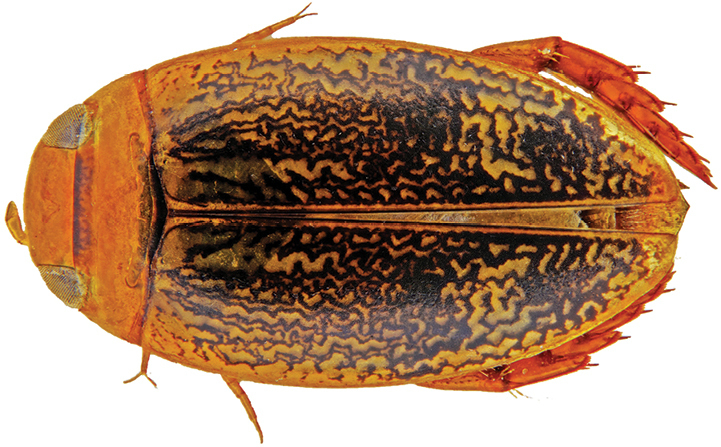
*Laccophilus simplicistriatus* Gschwendtner, 1932 3.8 mm, iSimangaliso Wetland Park, Eastern Shores (site 21), February 2015 DT Bilton, MS Bird & R Perissinotto leg.

## Appendix 1

Annotated and illustrated checklist of the Hydradephaga recorded from wetlands of the Lake St Lucia system, 2013–2015.

The following list includes photographs of all species recorded during the dedicated water beetle surveys conducted by the authors during the period 2013 to 2015. With the exception of *Laccophilus australis* Biström, Nilsson & Bergsten, 2015, this includes all species reliably recorded from the region.

### Family: Gyrinidae

#### Gyrinus natalensis

Taxon classification AnimaliaColeopteraGyrinidae

Régimbart, 1892

##### Remarks.

Standing waters.

##### Distribution.

Widespread to Western, Central and Eastern Africa.

##### St Lucia records.

Previously recorded by Vrdoljak (2004) in 2002/2003 at fresh water wetlands. Recorded at Western Shores in July 2014, during the course of this study.

#### Dineutus subspinosus

Taxon classification AnimaliaColeopteraGyrinidae

(Klug, 1834)

##### Remarks.

Standing waters.

##### Distribution.

Widespread to Western, Central and Eastern Africa.

##### St Lucia records.

Previously recorded by Vrdoljak (2004) in 2002/2003 at fresh water wetlands. Recorded at Eastern Shores and False Bay in January/February 2015, during the course of this study.

### Family: Haliplidae

#### Haliplus natalensis

Taxon classification AnimaliaColeopteraHaliplidae

Wehncke, 1880

##### Remarks.

Standing water, possibly associated with charophytes

##### Distribution.

Widespread to Western, Central and Eastern Africa

##### St Lucia records.

Not previously recorded from St Lucia. Recorded at False Bay in January/February 2015, during the course of this study.

#### Peltodytes sp.

Taxon classification AnimaliaColeopteraHaliplidae

##### Remarks.

This may be a new species, as only *Peltodytes quadratus* Régimbart, 1895 is recorded from South Africa (perhaps in error – see van Vondel, 2010) and this does not match the specimens collected. Only two females were found during this survey, making either positive identification or description impossible at present.

##### Distribution.

Unknown.

##### St Lucia records.

Not previously recorded from St Lucia. Recorded at Western Shores and Eastern Shores in January/February 2015, during the course of this study.

### Family: Noteridae

#### Canthydrus apicicornis

Taxon classification AnimaliaColeopteraNoteridae

Régimbart, 1895

##### Remarks.

Standing waters, in dense vegetation.

##### Distribution.

Kwa-Zulu Natal and Mozambique. Endemic to South-East Africa.

##### St Lucia records.

Not previously recorded from St Lucia. Recorded at Western Shores and Eastern Shores in July 2014 and January/February 2015, during the course of this study.

#### Canthydrus marshalli

Taxon classification AnimaliaColeopteraNoteridae

Balfour-Browne, 1939

##### Remarks.

Standing waters, in dense vegetation.

##### Distribution.

Kwa-Zulu Natal and Central Africa.

##### St Lucia records.

Not previously recorded from St Lucia. Recorded at Western Shores in January/February 2015, during the course of this study.

#### Canthydrus notula

Taxon classification AnimaliaColeopteraNoteridae

(Erichson, 1843)

##### Remarks.

Standing waters, in dense vegetation.

##### Distribution.

Widespread throughout Africa.

##### St Lucia records.

Previously recorded by Vrdoljak (2004) in 2002/2003 in fresh water wetlands. Recorded at Western Shores, Eastern Shores and False Bay in July 2014 and January/February 2015, during the course of this study.

#### Canthydrus quadrivittatus

Taxon classification AnimaliaColeopteraNoteridae

(Boheman, 1848)

##### Remarks.

Standing waters, in dense vegetation.

##### Distribution.

Widespread to Central Africa.

##### St Lucia records.

Not previously recorded from St Lucia. Recorded at Western Shores, Eastern Shores and False Bay in July 2014 and January/February 2015, during the course of this study.

#### Canthydrus sedilloti

Taxon classification AnimaliaColeopteraNoteridae

Régimbart, 1895

##### Remarks.

Standing waters, in dense vegetation.

##### Distribution.

Widespread to Western, Central and East Africa.

##### St Lucia records.

Not previously recorded from St Lucia. Recorded at Western Shores and Eastern Shores in January/February 2015, during the course of this study.

#### Hydrocanthus grandis

Taxon classification AnimaliaColeopteraNoteridae

(Laporte, 1835)

##### Remarks.

Standing waters, in dense vegetation.

##### Distribution.

Botswana, Zimbabwe, Mozambique and to Central and Eastern Africa. New record for South Africa.

##### St Lucia records.

Not previously recorded from St Lucia. Recorded at Eastern Shores in January/February 2015, during the course of this study.

#### Hydrocanthus micans

Taxon classification AnimaliaColeopteraNoteridae

Wehncke, 1883

##### Remarks.

Standing waters, in dense vegetation.

##### Distribution.

Eastern Cape, Kwa-Zulu Natal, Botswana, Mozambique, Zimbabwe, Zambia and to Western, Central and Eastern Africa.

##### St Lucia records.

Not previously recorded from St Lucia. Recorded at Western Shores, Eastern Shores and False Bay in January/February 2015, during the course of this study.

#### Hydrocanthus ferruginicollis

Taxon classification AnimaliaColeopteraNoteridae

Régimbart, 1865

##### Remarks.

Fresh water bodies.

##### Distribution.

South Africa, Botswana, Zimbabwe, Mozambique to Central and Eastern Africa.

##### St Lucia records.

Previously recorded by Day et al. (1954) and Millard and Broekhuysen (1970) in 1964/1965 in fresh water streams feeding into South Lake. Recorded at False Bay in November 2013, during the course of this study.

#### Synchortus imbricatus

Taxon classification AnimaliaColeopteraNoteridae

(Klug, 1853)

##### Remarks.

Standing waters, in dense vegetation.

##### Distribution.

Kwa-Zulu Natal, Mozambique to Western, Central and Eastern Africa.

##### St Lucia records.

Not previously recorded from St Lucia. Recorded at Western Shores and Eastern Shores in January/February 2015, during the course of this study.

#### Synchortus desaegeri

Taxon classification AnimaliaColeopteraNoteridae

Gschwendtner, 1935

##### Remarks.

Standing waters, in dense vegetation.

##### Distribution.

Botswana to Central and Eastern Africa. New record for South Africa.

##### St Lucia records.

Not previously recorded from St Lucia. Recorded at Eastern Shores in January/February 2015, during the course of this study.

#### Neohydrocoptus aethiopicus

Taxon classification AnimaliaColeopteraNoteridae

(Balfour-Browne, 1961)

##### Remarks.

Dense vegetation.

##### Distribution.

Western Cape to Central and Eastern Africa.

##### St Lucia records.

Not previously recorded from St Lucia. Recorded at Western Shores, Eastern Shores and False Bay in July 2014 and January/February 2015, during the course of this study.

#### Neohydrocoptus angolensis

Taxon classification AnimaliaColeopteraNoteridae

(Peschet, 1925)

##### Remarks.

Dense vegetation.

##### Distribution.

Kwa-Zulu Natal to Western and Eastern Africa.

##### St Lucia records.

Not previously recorded from St Lucia. Recorded at Western Shores, Eastern Shores and False Bay in July 2014 and January/February 2015, during the course of this study.

### Family: Dytiscidae

#### Copelatus cf. ejactus

Taxon classification AnimaliaColeopteraDytiscidae

Omer-Cooper, 1965

##### Remarks.

Thought to occur largely in standing waters. Possibly endemic to South Africa.

##### Distribution.

Previously known only from the Limpopo province.

##### St Lucia records.

Not previously recorded from St Lucia. Recorded at Western Shores, Eastern Shores and False Bay in January/February 2015, during the course of this study.

#### Copelatus erichsoni

Taxon classification AnimaliaColeopteraDytiscidae

Guérin-Méneville, 1849

##### Synonyms.

*Copelatus formosus* Wollaston, 1867.

##### Remarks.

Standing waters, especially shallow pools. Likely to be a complex of closely related species.

##### Distribution.

Widespread to Western, Central and Eastern Africa.

##### St Lucia records.

Not previously recorded from St Lucia. Recorded at Western Shores, Eastern Shores and False Bay in January/February 2015, during the course of this study.

#### Copelatus pulchellus

Taxon classification AnimaliaColeopteraDytiscidae

(Klug, 1834)

##### Synonyms.

*Copelatus africanus* Sharp, 1882; *Copelatus basalis* Boheman, 1848; *Copelatus discoideus* Sharp, 1882; *Colymbetes marginipennis* Laporte, 1835; *Copelatus obtusus* Boheman, 1848; *Copelatus strigulosus* Sharp, 1882.

##### Remarks.

Standing waters, especially shallow pools.

##### Distribution.

Widespread to Western and Eastern Africa.

##### St Lucia records.

Not previously recorded from St Lucia. Recorded at Eastern Shores and False Bay in January/February 2015, during the course of this study.

#### Cybister gschwendtneri

Taxon classification AnimaliaColeopteraDytiscidae

Guignot, 1935

##### Remarks.

Ponds and lagoons.

##### Distribution.

Widespread to Western and Eastern Africa, but not known to be common.

##### St Lucia records.

Not previously recorded from St Lucia. Recorded at Eastern Shores and False Bay in January/February 2015, during the course of this study.

#### Cybister marginicollis

Taxon classification AnimaliaColeopteraDytiscidae

Boheman, 1848

##### Synonyms.

*Cybister auritus* Gerstaecker, 1871; *Cybister filicornis* Sharp, 1882; *Cybister marginicollis annulicornis* Griffini, 1892.

##### Remarks.

Ponds and lagoons.

##### Distribution.

Widespread to Western, Central and Eastern Africa.

##### St Lucia records.

Previously recorded by Vrdoljak (2004) in 2002/2003 in fresh water wetlands. Recorded at Western Shores, Eastern Shores and False Bay in November 2013, July 2014 and January/February 2015, during the course of this study.

#### Cybister natalensis

Taxon classification AnimaliaColeopteraDytiscidae

(Wehncke, 1876)

##### Synonyms.

*Cybister circumcinctus* Gschwendtner, 1932

##### Remarks.

Ponds and lagoons.

##### Distribution.

Widespread to Central Africa.

##### St Lucia records.

Not previously recorded from St Lucia. Recorded at False Bay in January/February 2015, during the course of this study.

#### Cybister senegalensis

Taxon classification AnimaliaColeopteraDytiscidae

Aubé, 1838

##### Synonyms.

*Cybister convexiusculus* H.J. Kolbe, 1883; *Cybister marginellus* Régimbart, 1895; *Cybister rufiventris* Régimbart, 1895; Cybister senegalensis var. irroratus H.J. Kolbe, 1883; Cybister senegalensis var. seidlitzii Ragusa, 1888.

##### Remarks.

Ponds and lagoons.

##### Distribution.

Widespread to Western, Central and Eastern Africa.

##### St Lucia records.

Not previously recorded from St Lucia. Recorded at Western Shores and False Bay in January/February 2015, during the course of this study.

#### Cybister tripunctatus africanus

Taxon classification AnimaliaColeopteraDytiscidae

Laporte, 1835

##### Synonyms.

*Cybister aegyptiacus* Peyron, 1856; *Trogus haagi* Wehncke, 1876; *Trochalus meridionalis* Gené, 1836; *Trogus punctipennis* Taschenberg, 1883.

##### Remarks.

Abundant in ponds and lagoons.

##### Distribution.

Widespread to Mediterranean basin.

##### St Lucia records.

Not previously recorded from St Lucia. Recorded at Eastern Shores and False Bay in January/February 2015, during the course of this study.

#### Cybsiter bimaculatus

Taxon classification AnimaliaColeopteraDytiscidae

Aubé, 1838

##### Synonyms.

*Cybister aequatorius* Zimmermann, 1917; *Cybister regimbarti* Wilke, 1920.

##### Remarks.

Ponds and lagoons.

##### Distribution.

Widespread in Africa.

##### St Lucia records.

Not previously recorded from St Lucia. Recorded at Eastern Shores in January/February 2015, during the course of this study.

#### Cybister ertli

Taxon classification AnimaliaColeopteraDytiscidae

Zimmermann, 1917

##### Remarks.

Ponds and lagoons.

##### Distribution.

Swaziland, Zimbabwe, Mozambique, Malawi to Central and Eastern Africa. New record for South Africa.

##### St Lucia records.

Not previously recorded from St Lucia. Recorded at Eastern Shores in January/February 2015, during the course of this study.

#### Cybister vicinus

Taxon classification AnimaliaColeopteraDytiscidae

Zimmermann, 1917

##### Remarks.

Ponds and lagoons.

##### Distribution.

Mpumalanga and widespread to Central and Eastern Africa.

##### St Lucia records.

Not previously recorded from St Lucia. Recorded at Eastern Shores and False Bay in January/February 2015, during the course of this study.

#### Cybister vulneratus

Taxon classification AnimaliaColeopteraDytiscidae

Klug, 1834

##### Synonyms.

*Cybister binotatus* Klug, 1835; *Cybister bivuolnerus* Aubé, 1838; *Cybister madagascariensis* Aubé, 1838.

##### Remarks.

Ponds and lagoons.

##### Distribution.

Widespread to Mediterranean basin.

##### St Lucia records.

Previously recorded by Vrdoljak (2004) in 2002/2003 at fresh water wetlands. Recorded at Western Shores, Eastern Shores and False Bay in November 2013, July 2014 and January/February 2015, during this study.

#### Rhantaticus congestus

Taxon classification AnimaliaColeopteraDytiscidae

(Klug, 1833)

##### Synonyms.

*Hydaticus rochasi* Perroud & Montrousier, 1864; *Hydaticus signatipennis* Laporte, 1835

##### Remarks.

Likely to be a species complex.

##### Distribution.

Widespread in Old World tropics.

##### St Lucia records.

Obtained from available museum collections and ad hoc collections by the University of KwaZulu-Natal in 2012. Recorded at Eastern Shores and False Bay in November 2013 and January/February 2015, during this study.

#### Eretes sticticus

Taxon classification AnimaliaColeopteraDytiscidae

(Linnaeus, 1767)

##### Synonyms.

*Eunectes helvolus* Klug, 1834; *Eunectes punctatus* Zoubkoff, 1837; *Eunectes occidentalis* Erichson, 1847; *Eunectes conicollis* Wollaston, 1861; *Eunectes subcoriaceus* Wollaston, 1861; *Eretes subdiaphanus* Wollaston, 1861.

##### Remarks.

Open ponds with bare substrate.

##### Distribution.

Widespread in Afrotropics, Middle East to Americas.

##### St Lucia records.

Not previously recorded from St Lucia. Recorded at Eastern Shores in January/February 2015, during the course of this study.

#### Hydaticus bivittatus

Taxon classification AnimaliaColeopteraDytiscidae

Laporte, 1835

##### Synonyms.

Hydaticus bivittatus var. sharpi Peschet, 1917.

##### Remarks.

Ponds.

##### Distribution.

Widespread to Western, Central and Eastern Africa.

##### St Lucia records.

Obtained from available museum collections and ad hoc collections by the South African National Collection of Insects. The year is not specified. Specimen found at the St Lucia lake body and immediate surrounds. Recorded at False Bay in January/February 2015, during the course of this study.

#### Hydaticus exclamationis

Taxon classification AnimaliaColeopteraDytiscidae

Aubé, 1838

##### Remarks.

Ponds.

##### Distribution.

Widespread to Western, Central and Eastern Africa.

##### St Lucia records.

Not previously recorded from St Lucia. Recorded at False Bay in January/February 2015, during the course of this study.

#### Hydaticus flavolineatus

Taxon classification AnimaliaColeopteraDytiscidae

Boheman, 1848

##### Remarks.

Ponds.

##### Distribution.

Widespread to Western, Central and Eastern Africa.

##### St Lucia records.

Not previously recorded from St Lucia. Recorded at False Bay in January/February 2015, during the course of this study.

#### Hydaticus cf. natalensis

Taxon classification AnimaliaColeopteraDytiscidae

Guignot, 1951

##### Remarks.

Ponds.

##### Distribution.

The identity of this species is currently uncertain, and will require comparisons with type specimens. Apparently endemic to KwaZulu-Natal.

##### St Lucia records.

Not previously recorded from St Lucia. Recorded at False Bay in January/February 2015, during the course of this study.

#### Hydaticus matruelis

Taxon classification AnimaliaColeopteraDytiscidae

Clark, 1864

##### Synonyms.

Hydaticus matruelis var. fuscicollis Régimbart, 1895; *Hydaticus graueri* Ahlwarth, 1921.

##### Remarks.

Ponds.

##### Distribution.

Widespread to Western, Central and Eastern Africa.

##### St Lucia records.

Not previously recorded from St Lucia. Recorded at False Bay in January/February 2015, during the course of this study.

#### Hydaticus servillianus

Taxon classification AnimaliaColeopteraDytiscidae

Aubé, 1838

##### Synonyms.

*Hydaticus discoidalis* Hope, 1843; *Hydaticus flavomarginatus* Zimmermann, 1920.

##### Remarks.

Ponds.

##### Distribution.

Widespread to Western, Central and Eastern Africa.

##### St Lucia records.

Not previously recorded from St Lucia. Recorded at Western Shores, Eastern Shores and False Bay in November 2013, July 2014 and January/February 2015, during the course of this study.

#### Bidessus sharpi

Taxon classification AnimaliaColeopteraDytiscidae

Régimbart, 1895

##### Synonyms.

*Bidessus factor* Omer-Cooper, 1959; *Bidessus granulum* Régimbart, 1859; *Bidessus sharpi nigeriensis* Omer-Cooper, 1974; *Bidessus sedilloti* Régimbart, 1859; *Bidessus sharpi sudanensis* Omer-Cooper, 1974.

##### Remarks.

Ponds, in shallow water with dense vegetation.

##### Distribution.

Widespread to Western, Central and Eastern Africa.

##### St Lucia records.

Not previously recorded from St Lucia. Recorded at Western Shores, Eastern Shores and False Bay in January/February 2015, during the course of this study.

#### Clypeodytes meridionalis

Taxon classification AnimaliaColeopteraDytiscidae

Régimbart, 1895

##### Synonyms.

*Clypeodytes seminulum* Régimbart, 1895; Clypeodytes cribrosus var. voiensis Guignot, 1936.

##### Remarks.

Ponds, in shallow water with dense vegetation.

##### Distribution.

Widespread to Western, Central and Eastern Africa.

##### St Lucia records.

Not previously recorded from St Lucia. Recorded at Western Shores and Eastern Shores in July 2014 and January/February 2015, during the course of this study.

#### Hydroglyphus farquharensis

Taxon classification AnimaliaColeopteraDytiscidae

(Scott, 1912)

##### Synonyms.

*Guignotus bivirgatus* Guignot, 1952; *Guignotus browni* Pederzani, 1982; *Guignotus harrisoni* Omer-Cooper, 1955.

##### Remarks.

Ponds.

##### Distribution.

Widespread to Eastern Africa.

##### St Lucia records.

Not previously recorded from St Lucia. Recorded at Western Shores, Eastern Shores and False Bay in January/February 2015, during the course of this study.

#### Hydroglyphus lineolatus

Taxon classification AnimaliaColeopteraDytiscidae

(Boheman, 1848)

##### Remarks.

Ponds, over exposed substrates.

##### Distribution.

Widespread in Southern Africa.

##### St Lucia records.

Not previously recorded from St Lucia. Recorded at Eastern Shores and False Bay in January/February 2015, during the course of this study.

#### Hydroglyphus zanzibarensis

Taxon classification AnimaliaColeopteraDytiscidae

(Régimbart, 1906)

##### Synonyms.

*Bidessus orarius* Omer-Cooper, 1931.

##### Remarks.

Ponds.

##### Distribution.

Widespread in Southern and Eastern Africa.

##### St Lucia records.

Not previously recorded from St Lucia. Recorded at Western Shores, Eastern Shores and False Bay in July 2014 and January/February 2015, during the course of this study.

#### Leiodytes hieroglyphicus

Taxon classification AnimaliaColeopteraDytiscidae

(Régimbart, 1894)

##### Synonyms.

*Clypeodytes ignobilis* Omer-Cooper, 1962; *Clypeodytes inumbratus* Guignot, 1936; *Clypeodytes lautus* Régimbart, 1895; *Clypeodytes ovatus* Omer-Cooper, 1931

##### Remarks.

Ponds.

##### Distribution.

Widespread to Western, Central and Eastern Africa.

##### St Lucia records.

Not previously recorded from St Lucia. Recorded at Western Shores and Eastern Shores in January/February 2015, during the course of this study.

#### Pseuduvarus viticollis

Taxon classification AnimaliaColeopteraDytiscidae

(Boheman, 1848)

##### Synonyms.

*Amarodytes octoguttatus caligosus* Guignot, 1946; *Bidessus gentilis* Sharp, 1890; *Uvarus monticola* Guignot, 1957; *Bidessus octoguttatus* Régimbart, 1895; *Bidessus ornatipennis* Régimbart, 1899.

##### Remarks.

Ponds.

##### Distribution.

Widespread to Western and Eastern Africa.

##### St Lucia records.

Not previously recorded from St Lucia. Recorded at Eastern Shores in January/February 2015, during the course of this study.

#### Uvarus gschwendtneri

Taxon classification AnimaliaColeopteraDytiscidae

(Guignot, 1942)

##### Synonyms.

*Bidessus opacus* Gschwendtner, 1935

##### Remarks.

Ponds.

##### Distribution.

Widespread to Central and Eastern Africa.

##### St Lucia records.

Not previously recorded from St Lucia. Recorded at Western Shores, Eastern Shores and False Bay in January/February 2015, during the course of this study.

#### Hydrovatus acuminatus

Taxon classification AnimaliaColeopteraDytiscidae

Motschulsky, 1859

##### Synonyms.

*Hydrovatus acuminatus furvus* Guignot, 1950; *Hydrovatus affinis* Régimbart, 1895; *Hydroporus badius* Clark, 1863; *Hydrovatus consanguineous* Régimbart, 1880; *Hydrovatus ferrugineus* Zimmermann, 1919; *Hydrovatus humilis* Sharp, 1882; *Hydroporus malaccae* Clark, 1863; *Hydrovatus obscurus* Motschulsky, 1859; *Hydrovatus obscurus* Régimbart, 1895; *Hydrovatus sordidus* Sharp, 1882

##### Remarks.

Ponds in dense vegetation.

##### Distribution.

Widespread in Afrotropics, to Oriental Region.

##### St Lucia records.

Not previously recorded from St Lucia. Recorded at Western Shores, Eastern Shores and False Bay in January/February 2015, during the course of this study.

#### Hydrovatus cribatus

Taxon classification AnimaliaColeopteraDytiscidae

Sharp, 1882

##### Synonyms.

*Hydrovatus dyscheres* Guignot, 1955; *Hydrovatus laticornis* Régimbart, 1895.

##### Remarks.

Ponds in dense vegetation.

##### Distribution.

Widespread to Western, Central and Eastern Africa.

##### St Lucia records.

Not previously recorded from St Lucia. Recorded at Western and Eastern Shores in January/February 2015, during the course of this study.

#### Hydrovatus dentatus

Taxon classification AnimaliaColeopteraDytiscidae

Bilardo & Rocchi, 1990

##### Remarks.

Ponds in dense vegetation.

##### Distribution.

KwaZulu-Natal and Zambia. Appears to be rare.

##### St Lucia records.

Not previously recorded from St Lucia. Recorded at Western Shores and Eastern Shores in January/February 2015, during the course of this study.

#### Hydrovatus eximius

Taxon classification AnimaliaColeopteraDytiscidae

Biström, 1997

##### Remarks.

Ponds in dense vegetation.

##### Distribution.

Zimbabwe and Mozambique. New record for South Africa.

##### St Lucia records.

Not previously recorded from St Lucia. Recorded at False Bay in January/February 2015, during the course of this study.

#### Hydrovatus nefandus

Taxon classification AnimaliaColeopteraDytiscidae

Omer-Cooper, 1957

##### Remarks.

Ponds in dense vegetation.

##### Distribution.

Widespread in Southern Africa.

##### St Lucia records.

Not previously recorded from St Lucia. Recorded at Eastern Shores and False Bay in January/February 2015, during the course of this study.

#### Hydrovatus nigricans

Taxon classification AnimaliaColeopteraDytiscidae

Sharp, 1882

##### Synonyms.

*Hydrovatus abotti* Guignot, 1959.

##### Remarks.

Ponds in dense vegetation.

##### Distribution.

Widespread to Central and Eastern Africa.

##### St Lucia records.

Not previously recorded from St Lucia. Recorded at Eastern Shores in January/February 2015, during the course of this study.

#### Hydrovatus oblongipennis

Taxon classification AnimaliaColeopteraDytiscidae

Régimbart, 1895

##### Synonyms.

*Hydrovatus crassus* Guignot, 1958.

##### Remarks.

Ponds in dense vegetation.

##### Distribution.

Widespread to Western, Central and Eastern Africa.

##### St Lucia records.

Not previously recorded from St Lucia. Recorded at Eastern Shores in July 2014 and January/ February 2015, during the course of this study.

#### Hydrovatus obsoletus

Taxon classification AnimaliaColeopteraDytiscidae

Peschet, 1922

##### Synonyms.

*Hydrovatus adelphus* Guignot, 1956; *Hydrovatus straeleni* Guignot, 1947.

##### Remarks.

Ponds in dense vegetation.

##### Distribution.

Widespread to Central and Eastern Africa.

##### St Lucia records.

Not previously recorded from St Lucia. Recorded at Eastern Shores in January/February 2015, during the course of this study.

#### Hydrovatus villiersi

Taxon classification AnimaliaColeopteraDytiscidae

Guignot, 1955

##### Synonyms.

*Hydrovatus albertianus* Guignot, 1959.

##### Remarks.

Ponds in dense vegetation.

##### Distribution.

Widespread to Western, Central and Eastern Africa.

##### St Lucia records.

Not previously recorded from St Lucia. Recorded at Eastern Shores in January/February 2015, during the course of this study.

#### Hydrovatus visendus

Taxon classification AnimaliaColeopteraDytiscidae

Biström, 1997

##### Remarks.

Ponds in dense vegetation.

##### Distribution.

Zimbabwe to Eastern Africa. New record for South Africa.

##### St Lucia records.

Not previously recorded from St Lucia. Recorded at Western Shores in January/ February 2015, during the course of this study.

#### Herophydrus guineensis

Taxon classification AnimaliaColeopteraDytiscidae

(Aubé, 1838)

##### Synonyms.

*Hydroporus barbarous* Schaum, 1847; *Hydroporus ferrugineus* Lucas, 1846; *Hydroporus hyphydroides* Perris, 1864; *Hydroporus inflatus* Reiche, 1869; *Hydroporus turgidus* Erichson, 1843; *Herophydrus umbrosus* Zimmermann, 1926.

##### Remarks.

Ponds.

##### Distribution.

Widespread to Western, Central and Eastern Africa.

##### St Lucia records.

Not previously recorded from St Lucia. Recorded at Western Shores, Eastern Shores and False Bay in January/February 2015, during the course of this study.

#### Herophydrus inquinatus

Taxon classification AnimaliaColeopteraDytiscidae

(Boheman, 1848)

##### Synonyms.

*Herophydrus cooperi* Gschwendtner, 1938; *Herophydrus ignoratus* Gschwendtner, 1933; *Herophydrus kalaharii* Gschwendtner, 1935.

##### Remarks.

Ponds and stream pools. Eurytopic.

##### Distribution.

Widespread to Central and Eastern Africa.

##### St Lucia records.

Not previously recorded from St Lucia. Recorded at False Bay in November 2013 and January/February 2015, during the course of this study.

#### Herophydrus nigrescens

Taxon classification AnimaliaColeopteraDytiscidae

Biström & Nilsson, 2002

##### Remarks.

Ponds.

##### Distribution.

Endemic to KwaZulu-Natal.

##### St Lucia records.

Not previously recorded from St Lucia. Recorded at Western Shores, Eastern Shores and False Bay in January/February 2015, during the course of this study.

#### Herophydrus nodieri

Taxon classification AnimaliaColeopteraDytiscidae

(Régimbart, 1895)

##### Remarks.

Ponds.

##### Distribution.

Widespread to Western, Central and Eastern Africa.

##### St Lucia records.

Not previously recorded from St Lucia. Recorded at Western Shores, Eastern Shores and False Bay in November 2013, July 2014 and January/February 2015, during the course of this study.

#### Heterhydrus senegalensis

Taxon classification AnimaliaColeopteraDytiscidae

(Laporte, 1835)

##### Remarks.

Ponds.

##### Distribution.

Widespread to Western, Central and Eastern Africa.

##### St Lucia records.

Not previously recorded from St Lucia. Recorded at Western Shores and Eastern Shores in January/February 2015, during the course of this study.

#### Hyphydrus caffer

Taxon classification AnimaliaColeopteraDytiscidae

Boheman, 1848

##### Remarks.

Ponds.

##### Distribution.

Widespread to Central and Eastern Africa.

##### St Lucia records.

Not previously recorded from St Lucia. Recorded at Eastern Shores in January/February 2015, during the course of this study.

#### Hyphydrus cycloides

Taxon classification AnimaliaColeopteraDytiscidae

Régimbart, 1889

##### Synonyms.

*Hyphydrus circularis* Régimbart, 1895; *Hyphydrus lamottei* Legros, 1958; *Hyphydrus malawiensis* Omer-Cooper, 1971; *Hyphydrus nigeriensis* Omer-Cooper, 1971; *Hyphydrus pelates* Guignot, 1953.

##### Remarks.

Ponds.

##### Distribution.

Widespread to Central and Eastern Africa.

##### St Lucia records.

Previously recorded by Vrdoljak (2004) in 2002/2003 at freshwater wetlands. Recorded at Western Shores, Eastern Shores and False Bay in January/February 2015, during this study.

#### Hyphydrus signatus

Taxon classification AnimaliaColeopteraDytiscidae

Sharp, 1882

##### Synonyms.

*Hyphydrus aethiopicus* J. Balfour-Browne, 1944; *Hyphydrus grossus* Sharp, 1882.

##### Remarks.

Ponds.

##### Distribution.

Widespread to central and Eastern Africa.

##### St Lucia records.

Previously recorded by Day et al (1954) and Millard and Broekhuysen (1970) in 1964/1965 at fresh water streams feeding into South Lake. Recorded at Western Shores and False Bay in November 2013 and January/February 2015, during this study.

#### Methles cribratellus

Taxon classification AnimaliaColeopteraDytiscidae

(Fairmaire, 1880)

##### Synonyms.

*Methles punctipennis* Sharp, 1882; *Methles umbrosus* Gschwendtner, 1930.

##### Remarks.

Ponds in dense vegetation.

##### Distribution.

Widespread to Mediterranean basin and Middle East.

##### St Lucia records.

Not previously recorded from St Lucia. Recorded at Western Shores, Eastern Shores and False Bay in July 2014 and January/February 2015, during this study.

#### Derovatellus cf. natalensis

Taxon classification AnimaliaColeopteraDytiscidae

Omer-Cooper, 1965

##### Remarks.

Ponds and small wetlands with dense vegetation. This beetle is either *Derovatellus natalensis* or an undescribed species. Omer-Cooper (1965) states that the type is in the Natural History Museum, London, but no specimens of this species are present in the collection (D.T. Bilton, *pers. obs.*). The male genitalia are close to Omer-Cooper’s figures of *natalensis*, but do differ. It is hoped that the identity of these specimens can be resolved by future studies of material named by Omer-Cooper.

##### Distribution.

Endemic to South-East Africa.

##### St Lucia records.

Not previously recorded from St Lucia. Recorded at Western Shores, Eastern Shores and False Bay in November 2013 and January/February 2015, during this study.

#### Laccophilus canthydroides

Taxon classification AnimaliaColeopteraDytiscidae

Omer-Cooper, 1957

##### Remarks.

Uncertain. Appears to prefer dense vegetation.

##### Distribution.

Described from South Africa. Now known to be widespread, from Cameroon and Ethiopia through east Africa to the Cape (Biström et al., 2015).

##### St Lucia records.

Not previously recorded from St Lucia. Recorded at Western Shores, Eastern Shores and False Bay in January/February 2015, during the course of this study.

#### Laccophilus cryptos

Taxon classification AnimaliaColeopteraDytiscidae

Biström, Nilsson & Bergsten, 2015

##### Remarks.

Ponds.

##### Distribution.

Widespread in Southern Central and Eastern Africa.

##### St Lucia records.

Holotype and some paratypes taken at light at St Lucia in 1975. Recorded at Western Shores, Eastern Shores and False Bay in July 2014 and January/February 2015, during the course of this study.

#### Laccophilus contiro

Taxon classification AnimaliaColeopteraDytiscidae

Guignot, 1952

##### Remarks.

Ponds and other small waterbodies.

##### Distribution.

Widespread to Western, Central and Eastern Africa.

##### St Lucia records.

Not previously recorded from St Lucia. Recorded at Western Shores, Eastern Shores and False Bay in January/February 2015, during the course of this study.

#### Laccophilus simplicistriatus

Taxon classification AnimaliaColeopteraDytiscidae

Gschwedntner, 1932

##### Synonyms.

*Laccophilus monas* Guignot, 1953.

##### Remarks.

Known from a range of habitats including a reservoir, river pools, river swamps, waterholes and dams (Biström et al., 2015).

##### Distribution.

Widespread from Sudan to South Africa and Namibia.

##### St Lucia records.

Not previously recorded from St Lucia. Recorded at Western Shores, Eastern Shores and False Bay in November 2013, July 2014 and January/February 2015, during the course of this study.
